# PARP1 UFMylation ensures the stability of stalled replication forks

**DOI:** 10.1073/pnas.2322520121

**Published:** 2024-04-24

**Authors:** Yamin Gong, Zhifeng Wang, Wen Zong, Ruifeng Shi, Wenli Sun, Sijia Wang, Bin Peng, Shunichi Takeda, Zhao-Qi Wang, Xingzhi Xu

**Affiliations:** ^a^Guangdong Key Laboratory for Genome Stability & Disease Prevention, Shenzhen University School of Medicine, Shenzhen, Guangdong 518060, China; ^b^Leibniz Institute on Aging–Fritz Lipmann Institute, Jena 07745, Germany; ^c^State Key Laboratory of Microbial Technology, Shandong University, Qingdao 266237, China; ^d^Faculty of Biology and Pharmacy, Friedrich-Schiller University of Jena, Jena 07743, Germany

**Keywords:** UFMylation, DNA replication, PARP1, replication stress

## Abstract

The S-phase checkpoint involving CHK1 is essential for fork stability in response to fork stalling. PARP1 acts as a sensor of replication stress and is required for CHK1 activation. However, it is unclear how the activity of PARP1 is regulated. Here, we found that UFMylation is required for the efficient activation of CHK1 by UFMylating PARP1 at K548 during replication stress. Inactivation of UFL1, the E3 enzyme essential for UFMylation, delayed CHK1 activation and inhibits nascent DNA degradation during replication blockage as seen in PARP1-deficient cells. An in vitro study indicated that PARP1 is UFMylated at K548, which enhances its catalytic activity. Correspondingly, a PARP1 UFMylation-deficient mutant (K548R) and pathogenic mutant (F553L) compromised CHK1 activation, the restart of stalled replication forks following replication blockage, and chromosome stability. Defective PARP1 UFMylation also resulted in excessive nascent DNA degradation at stalled replication forks. Finally, we observed that PARP1 UFMylation-deficient knock-in mice exhibited increased sensitivity to replication stress caused by anticancer treatments. Thus, we demonstrate that PARP1 UFMylation promotes CHK1 activation and replication fork stability during replication stress, thus safeguarding genome integrity.

## Significance

UFMylation, the latest addition to the ubiquitin-like posttranslational modifications, plays an important role in a variety of cellular activities. However, its potential role in DNA replication and replication stress response is unknown. This study uncovers that UFMylation is required for stabilization of stalled replication forks in response to DNA replication stress. Mechanistically, PARP1 UFMylation at K548 ensures activation of the replication checkpoint signaling and facilitates restart of stalled replication fork. Impaired PARP1 UFMylation results in genomic instability, and genetically modified mice with defective UFMylation exhibit increased sensitivity to replication stress.

To maintain genome integrity upon replication stalling at damaged template strands, cells have evolved a complex mechanism—the S-phase checkpoint—to detect and repair DNA damage that occurs during replication. This checkpoint is mediated by the ATR-CHK1 pathway (1–3), which detects DNA lesions and activates the cell cycle checkpoint to restore replication forks and ensure their progression.

PARP1 controls fork speed and the choice of stress response mechanisms (4–7). PARP1 also plays an important role in processing Okazaki fragments (8–11). PARP1 binds rapidly to various naked DNAs, including DNA gaps, double-strand break (DSB) ends, and blocked or collapsed replication forks (12–14). The binding immediately activates PARP1 and adds ADP-ribose to various substrates, but mainly itself at the middle automodification domain, which consists of a BRCT (BRCA1 C-terminal) and a WGR (tryptophan-glycine–arginine) domain. It is believed that autoPARylation or its product PAR recruits various effective factors to the damaged sites (15). Although WGR is outside of the catalytic core, it seems to participate in regulating PARylation, likely due to 3D folding to affect activity (12, 16). PARP1 is subjected to autoPARylation, ubiquitination, and SUMOylation, which facilitates the removal of PARP1 from DNA lesions when they trap PARP1 (17–20). It remains unclear whether any other posttranslational modifications significantly control the activity of PARP1.

UFM1 is a newly identified ubiquitin-like protein that covalently links to substrates in a process known as UFMylation. The enzymes involved in this process include the E1-like enzyme UBA5, the E2-like conjugase UFC1, and the E3-like ligase UFL1 (21, 22). The process of UFMylation is reversed by the deUFMylase UfSP1/2 (23, 24). UFMylation is involved in multiple cellular processes, such as endoplasmic reticulum stress, DSB repair, telomere maintenance, and genome stability (22, 25–28) However, whether UFMylation participates in the replication stress response is still unknown.

Here, we aimed to determine whether UFMylation is involved in the replication stress in order to understand the regulation of replication fork stability. We found that PARP1 is UFMylated at the WGR domain, enhancing its PARylation activity and subsequently promoting the activation of CHK1 and the recruitment of MRE11 at stalled replication forks. And the in vivo experiment showed that the UFMylation of PARP1 is important for genomic stability and genotoxic response, demonstrating the effects of PARP1 UFMylation in improving fork stability and repair and the impact on genome stability.

## Results

### Defective UFMylation Compromises Stalled Replication Fork Stability and Restart.

**Fig. 1.**
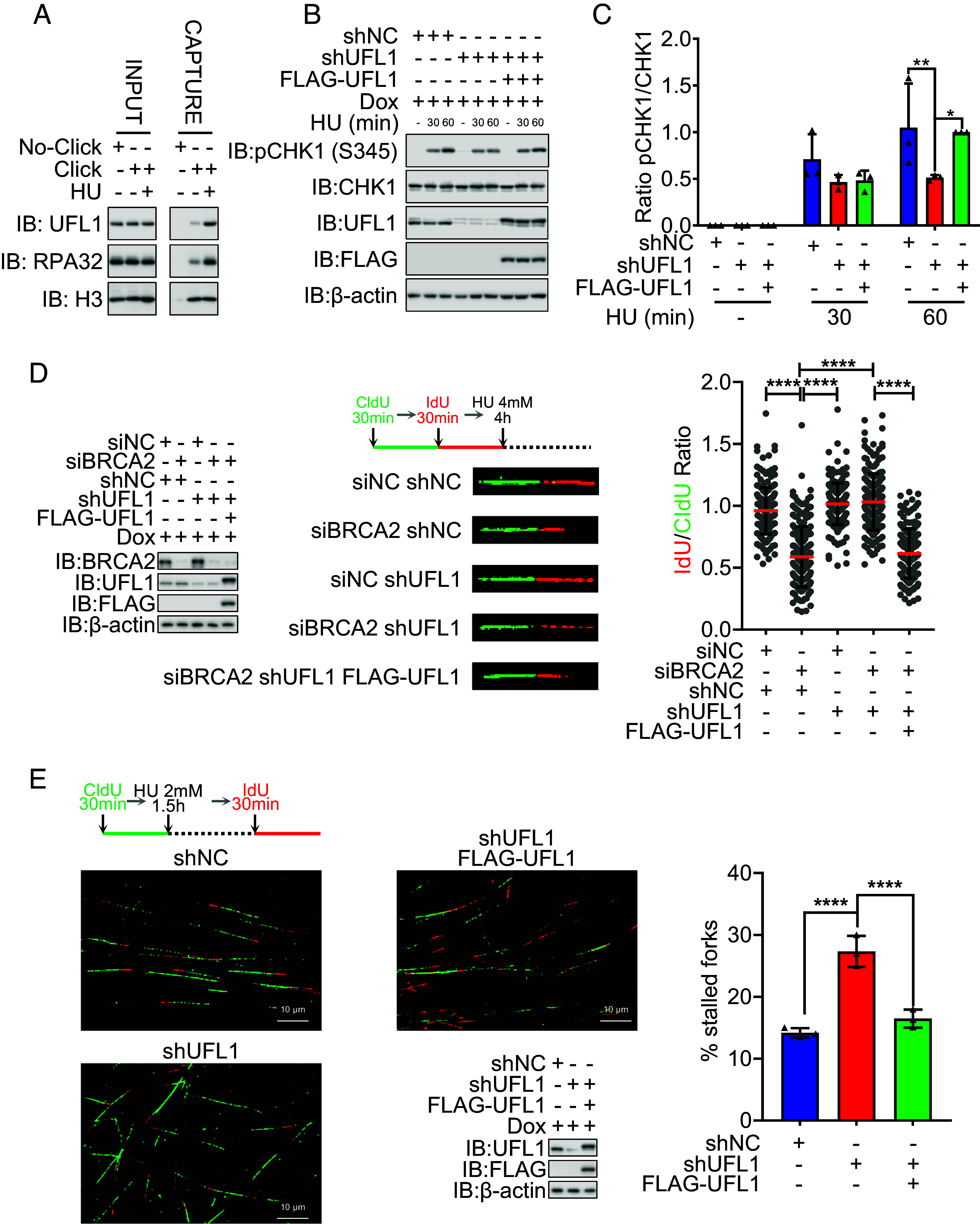
Defective UFMylation compromises stalled replication fork stability and restart. (*A*) UFL1 was recruited to the stalled replication forks. HeLa cells were treated with or without HU (4 mM, 4 h). Cells were harvested for iPOND, and the total cell lysates were subjected to IP-WB with the indicated antibodies. (*B* and *C*) UFL1 depletion compromised the activation of CHK1. The stable HeLa cell lines of shNC (negative control), shUFL1 (UFL1 depleted), and FLAG-UFL1 (shUFL1-resistant UFL1 cDNA reexpressed) treated with or without HU (2 mM) for the indicated times were harvested. The activation of CHK1 was analyzed by SDS-PAGE and WB (*B*) with indicated antibodies. (*C*) The respective quantifications for activation of CHK1 are shown, and a column plot with bar of the ratios of pCHK1 and CHK1 is shown. Two-way ANOVA (Tukey’s multiple comparisons test): *P < 0.05, **P < 0.01, N = 3. (*D*) UFL1 depletion compromised the degradation of stalled replication forks. The stable HeLa cell lines of shNC (negative control), shUFL1 (UFL1 depleted), and FLAG-UFL1 (shUFL1-resistant UFL1 cDNA reexpressed) were transfected with siBRCA2 and then labeled with CldU and then IdU, before HU treatment (4 mM, 4 h), and then, the DNA fiber assay was conducted to determine the degradation of stalled replication forks. At least 100 tracts were counted, and the experiment was repeated three times. Protein expression levels identified by WB (*Left*), representative images of CldU (green) and IdU (red) replication tracks (*Middle*), and a scatter plot of IdU/CldU tract length ratios (*Right*) for individual replication forks are shown. Unpaired *t* test (Mann–Whitney test): ****P < 0.0001. (*E*) UFL1 depletion compromised the restart of stalled replication forks. The stable HeLa cell lines of shNC (negative control), shUFL1 (UFL1 depleted), and FLAG-UFL1 (shUFL1-resistant UFL1 cDNA reexpressed) were labeled with CldU and then HU treatment (HU 2 mM for 1.5 h) and then labeled with IdU. The cells were harvested immediately, and the DNA fiber assay was conducted to determine the restart of stalled replication forks. At least 100 tracts were counted, and the experiment was repeated three times. Representative images of CldU (green) and IdU (red) replication tracks (*Left* and *Middle Up*), protein expression levels identified by WB (*Middle Bottom*), and a column plot with bar of the ratios of CldU without IdU tracts (*Right*) for replication forks are shown. Paired *t* test: ****P < 0.0001. IB: immunoblot; WB: western blotting; IP: immunoprecipitation; HU: hydroxyurea; Dox: doxycycline. H3 and β-actin were used as loading controls.

To explore the function of UFMylation at replication forks, we first conducted an iPOND assay to detect the factors associated with the replication forks and are involved in UFMylation. Indeed, upon treatment with hydroxyurea (HU) (4 mM, 4 h), a ribonucleotide reductase II inhibitor, which depletes cellular dNTP pools, arrests replication forks, RPA binding and UFM1 ligase UFL1 were cofound at replication forks (Fig. 1*A*). We next depleted UFL1 by using inducible stable knockdown cell line induced by doxycycline (Dox) and found that it reduced HU-induced activation of CHK1 (judged by p-Chk1), a key regulator of the S phase, which was restored by reexpression of shUFL1-resistant UFL1 cDNA (Fig. 1 *B* and *C* and *SI Appendix*, Fig. S1*A*). These findings indicate that UFMylation is involved in S-phase checkpoint activation.

We then conducted DNA fiber assays to analyze the nascent DNA resection and restart of stalled replication forks. The results showed that UFL1 depletion prevented the degradation of replication forks in BRCA2-deficient cells after 4 h of HU (4 mM) treatment (Fig. 1*D*) Additionally, it reduced the restart of stalled replication forks after 1.5 h of HU (2 mM) treatment (Fig. 1*E*). Both effects were restored by reexpressing UFL1 cDNA (Fig. 1 *D* and *E*). We thus concluded that UFMylation is important for stalled replication fork degradation and restart following replication stress.

### PARP1 Is UFMylated on K548.

**Fig. 2.**
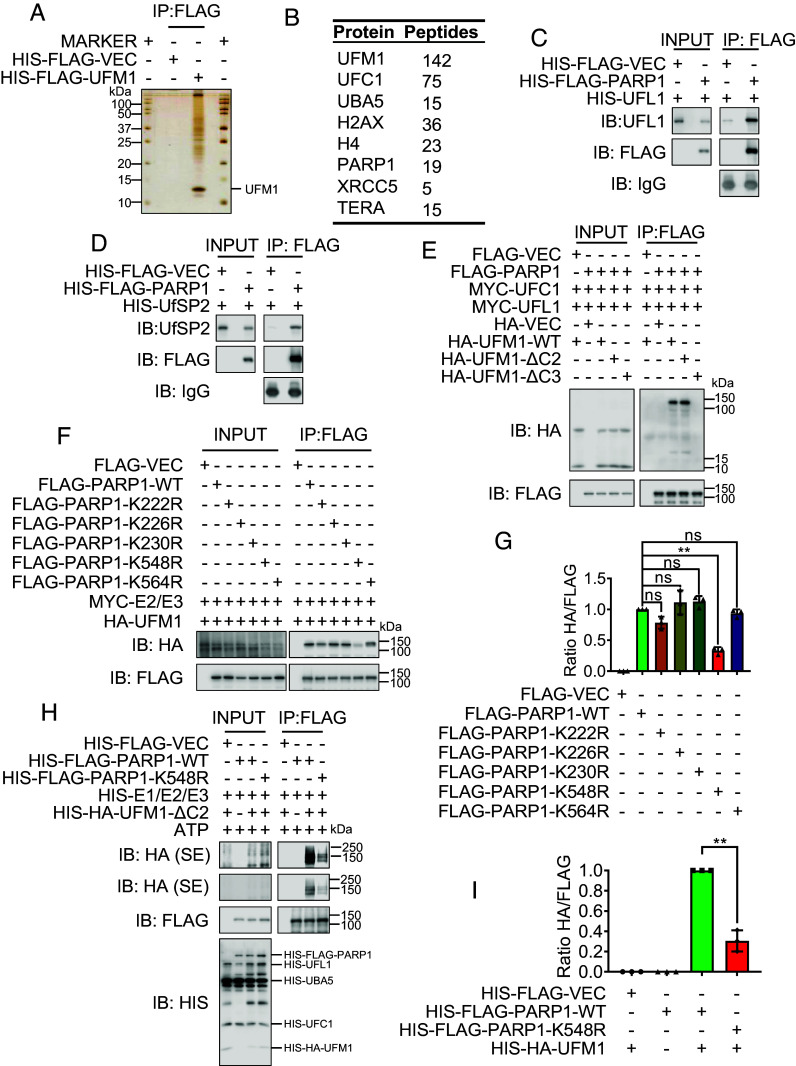
PARP1 is UFMylated on K548. (*A* and *B*) Identification of UFMylation substrates. FLAG-VEC and FLAG-UFM1 stable expressed HeLa cells were harvested and the lysates were subjected to denatured IP, and the FLAG-UFM1 were eluted with FLAG peptides. (*A*) The silver staining of IP complexes separated by SDS-PAGE. (*B*) The selected candidates of mass spectrometric analysis of the UFM1 immunoprecipitates. (*C* and *D*) PARP1 interacted with UFL1 and UfSP2 in vitro. Bacterially produced HIS-FLAG-VEC/PARP1 were used to pull down bacterially purified HIS-UFL1 (*C*) or HIS-UfSP2 (*D*). (*E*) PARP1 was UFMylated. FLAG-PARP1 or Vector, together with MYC-UFC1 and MYC-UFL1, was co-transfected into HEK293T cells with HA-UFM1-WT, HA-UFM1-ΔC2, HA-UFM1-ΔC3, or HA-Vector as indicated. The transfectants were lysed 48 h after transfection and subjected to denatured immunoprecipitation. The immunoprecipitates were analyzed by SDS-PAGE and WB with indicated antibodies. HA-UFM1-WT and HA-UFM1-ΔC2 are the active states of UFM1, and HA-UFM1-ΔC3 is the inactive state of UFM1. (*F* and *G*) The K548R mutation of PARP1 decreased its UFMylation. FLAG-PARP1 WT, K548R, or Vector, was co-transfected into HEK293T cells with HA-UFM1, MYC-UFC1 (E2), and MYC-UFL1 (E3). The UFMylation levels of PAPR1 were analyzed by SDS-PAGE and WB (*F*) with indicated antibodies. (*G*) The respective quantifications for PARP1 UFMylation are shown, and a column plot with bar of the ratios of HA and FLAG is shown. Paired *t* test: ns = nonsignificant; **P < 0.01, N = 3. (*H* and *I*) PARP1 was UFMylated on K548 in vitro. Recombinant UFMylation factors (HIS-UBA5, HIS-UFC1, HIS-UFL1, and HIS-HA-UFM1-ΔC2) with bacterially produced HIS-FLAG-VEC, PARP1, or K548R were incubated in UFMylation buffer at 30 °C for 90 min. The reaction was terminated by adding SDS sample buffer, and the samples were subjected to SDS-PAGE followed by WB (*H*) with the indicated antibodies. (*I*) The respective quantifications for PARP1 UFMylation are shown, and a column plot with bar of the ratios of HA and FLAG is shown. Paired *t* test: **P < 0.01, N = 3. IP: immunoprecipitation; IB: immunoblot; WB: western blotting; SE: short exposure; LE: long exposure.

To identify the mechanism by which UFMylation regulates replication fork stability, we performed a series of UFMylation assays to identify its substrate. First, we conducted a denatured immunoprecipitation (IP) of FLAG-UFM1 and analyzed the precipitated proteins by mass spectrometry (MS). Interestingly, we detected PARP1, which is involved in replication fork regulation (29, 30), on the top hits, in addition to UFMylation factors, of the protein list of more than 600 proteins (Fig. 2 *A* and *B*). We further studied whether PARP1 is a substrate of UFMylation. By IP, we found that PARP1 interacts with both the UFM1-specific ligase UFL1 and the UFM1-specific protease UfSP2 (*SI Appendix*, Fig. S2 *A* and *B*). These interactions were further confirmed in vitro using purified UFL1, UfSP2, and PARP1 from *Escherichia coli* (Fig. 2 *C* and *D*). Furthermore, the interaction between UFL1 and PARP1 was enhanced upon HU (2 mM) treatment (*SI Appendix*, Fig. S2*C*), suggesting that PARP1 UFMylation plays a role in replication stress response.

Next, PARP1 UFMylation was confirmed in vivo. We cotransfected HEK293T cells with FLAG-PARP1, MYC-UFC1, MYC-UFL1, and HA-UFM1 or HA-UFM1-ΔC2 or HA-UFM1-ΔC3 (negative controls). The results of the denaturing IP revealed a specific band of UFM1, represented by a protein signal approximately 10 kDa larger than that of PARP1. This band was not observed in the IP using the conjugation-defective UFM1-ΔC3 (deletion of three C-terminal amino acids including glycine 83). We thus presumed that there is a covalent linkage between UFM1 and PARP1 (Fig. 2*E*). This covalent link was up-regulated upon HU treatment (*SI Appendix*, Fig. S2*D*), which is similar to the interaction pattern between UFL1 and PARP1 (*SI Appendix*, Fig. S2*C*). These results suggest the potential roles of PARP1 UFMylation in response to replication stress.

We next determined the sites of PARP1 UFMylation using a series of PARP1 truncations and lysine-to-arginine mutants (Fig. 2 *F* and *G* and *SI Appendix*, Fig. S2 *E*–*G*). From here, we found that the PARP1 K548R mutation eliminated its major UFMylation signal (Fig. 2 *F* and *G* and *SI Appendix*, Fig. S2*G*), indicating that K548 is a prominent UFMylation site. We confirmed this finding by conducting an in vitro UFMylation assay (Fig. 2 *H* and *I*). Interestingly, F553L, a K548 neighboring mutation, which is associated with human lung carcinoma based on the Cancer Genome Atlas (TCGA) database revealed, also compromised PARP1 UFMylation (*SI Appendix*, Fig. S2*G*), suggesting that deficiency in PARP1 UFMylation may contribute to the development of cancer. Taken together, these findings that PARP1 can be UFMylated and that a human tumor-associated mutation in PARP1 (PARP1-F553L) compromises PARP1 UFMylation demonstrate that PARP1 UFMylation may be involved in tumorigenesis.

### PARP1 UFMylation Promotes Its PARylation Activity.

**Fig. 3.**
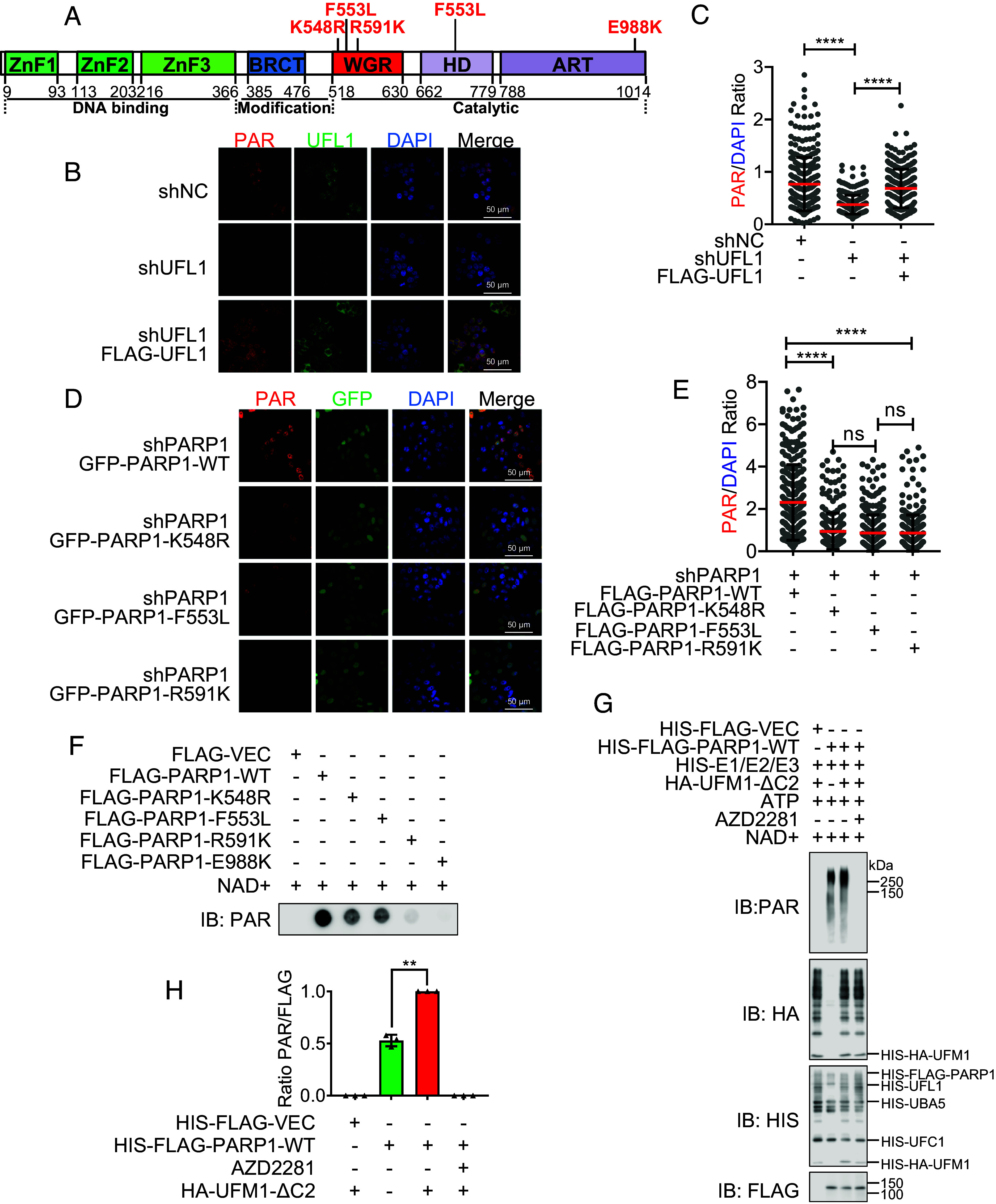
PARP1 UFMylation promotes its PARylation activity. (*A*) Schematic of human PARP1 domains and subdomains. The domains were shown as the zinc finger domains (ZnF) (green), BRCA C terminus (BRCT) (blue), a tryptophan–glycine–arginine (WGR) domain (red), and alpha-helical subdomain (HD) and ADP-ribosyl transferase subdomain (ART) (purple). (*B* and *C*) UFL1 depletion decreased the signal of PAR. The stable HeLa cell lines of shNC (negative control), shUFL1 (UFL1 depleted), and FLAG-UFL1 (shUFL1-resistant UFL1 cDNA reexpressed) were seeded on cover slips, and the PAR and UFL1 were detected with IF. (*B*) Representative images. (Scale bar: 50 μm.) (*C*) Scatter plot of PAR/DAPI relative intensity ratio. Unpaired *t* test (Mann–Whitney test): ns = nonsignificant; ****P < 0.0001. (*D* and *E*) PARP1 UFMylation and WGR domain are important for PAR formation. The stable HeLa cell lines of shPARP1 (PARP1 depleted) were transfected with GFP-PARP1 WT, K548R, F553L, or R591K, and the PAR was detected with IF after 24 h transfection. (*D*) Representative images. (Scale bar: 50 μm.) (*E*) Scatter plot of PAR/DAPI relative intensity ratio. Unpaired *t* test (Mann–Whitney test): ns = nonsignificant; ****P < 0.0001. (*F*) PARP1 UFMylation deficiency impaired the PAR formation. The in vitro PARylation assay was conducted with incubating HeLa cell derived FLAG-PARP1 WT, or K548R, or Vector with NAD^+^ at 30 °C for 30 min. The reaction was terminated by adding SDS sample buffer, and the samples were subjected to dot blot followed by immunoblotting with the indicated antibodies. (*G* and *H*) PARP1 UFMylation deficiency impaired its catalytic activity. Recombinant UFMylation factors (HIS-UBA5, HIS-UFC1, HIS-UFL1, and HIS-HA-UFM1-ΔC2) with bacterially produced HIS-FLAG-VEC, PARP1, or K548R were incubated in UFMylation buffer at 30 °C for 90 min. Then, 200 μM NAD^+^ was added and incubated at 30 °C for another 30 min. The reaction was terminated by adding SDS sample buffer, and the samples were subjected to SDS-PAGE followed by immunoblotting with the indicated antibodies. (*H*) The respective quantifications for PAR generation are shown, and a column plot with bar of the ratios of PAR and FLAG is shown. Paired *t* test: **P < 0.01, N = 3. IF: immunofluorescence; IP: immunoprecipitation; IB: immunoblot; AZD2281: PARP1 inhibitor.

The residue that we identified as essential for PARP1 UFMylation, K548, localizes in the PARP1 WGR domain, which is involved in PARylation activity due to its 3D conformation change, etc. (12) (Fig. 3*A*), indicating that PARP1 UFMylation may regulate PARP1 enzymatic activity. To investigate the influence of UFMylation on PARP1 activity, we first analyzed total PARylation in cells with UFL1 depletion. Immunofluorescence (IF) staining revealed that UFL1 depletion reduced total cellular PAR levels, which was restored upon reexpression of UFL1 (Fig. 3 *B* and *C* and *SI Appendix*, Fig. S3*A*). We confirmed these findings by immunoblotting, showing decreased cellular PAR levels in UFL1-deficient cells (*SI Appendix*, Fig. S3*C*). These results suggested that UFMylation is necessary for efficient PARylation.

Next, we examined the influence of PARP1 UFMylation on cellular PAR levels. To do so, we reexpressed GFP-tagged PARP1 in UFMylation-deficient mutants (GFP-PARP1-K548R and F553L) and a PARP1 WGR domain conservative mutant critical for PARP1 activation (GFP-PARP1-R591K) (31) in PARP1-depleted HeLa cells and analyzed cellular PAR levels. We observed decreased PARylation, judged by the PAR signal, compared with wild-type (WT) PARP1 cells (Fig. 3 *D* and *E* and *SI Appendix*, Fig. S3*B*). The results of an immunoblot assay in HeLa and MGC803 cells further confirmed that the PARP1-K548R mutant reduced total PAR levels (*SI Appendix*, Fig. S3 *D* and *E*). Moreover, HU exposure increased PARylation in the WT, but not mutant-reconstituted cells (*SI Appendix*, Fig. S3*E*). As a control, we treated these cells with the PARP1 inhibitor AZD2281 (olaparib) and observed that this inhibitor abolished cellular PARylation in both WT and mutant contexts (*SI Appendix*, Fig. S3*E*). These results indicate that UFMylation of PARP1 positively modulates the PARylation capacity of PARP1.

To exclude other factors that may influence PARP1 PARylation in vivo, we purified PARP1 WT and mutant (K548R/F553L/R591K) proteins from HeLa cells and performed an in vitro PARylation assay. The PARP1 mutants showed impaired PARylation compared with WT (Fig. 3*F* and *SI Appendix*, Fig. S3 *F*–*H*). We also performed an in vitro PARylation assay following in vitro UFMylation with bacterially produced PARP1 and UFMylation factors (UBA5, UFC1, UFL1, and UFM1). Here, PARP1 PARylation was increased when UFM1 was added to the experimental system (Fig. 3 *G* and *H*). We thus conclude that PARP1 UFMylation promotes its catalytic activity.

### Defective PARP1 UFMylation Impairs CHK1 Activation and MRE11 Recruitment to the Stalled Replication Forks.

**Fig. 4.**
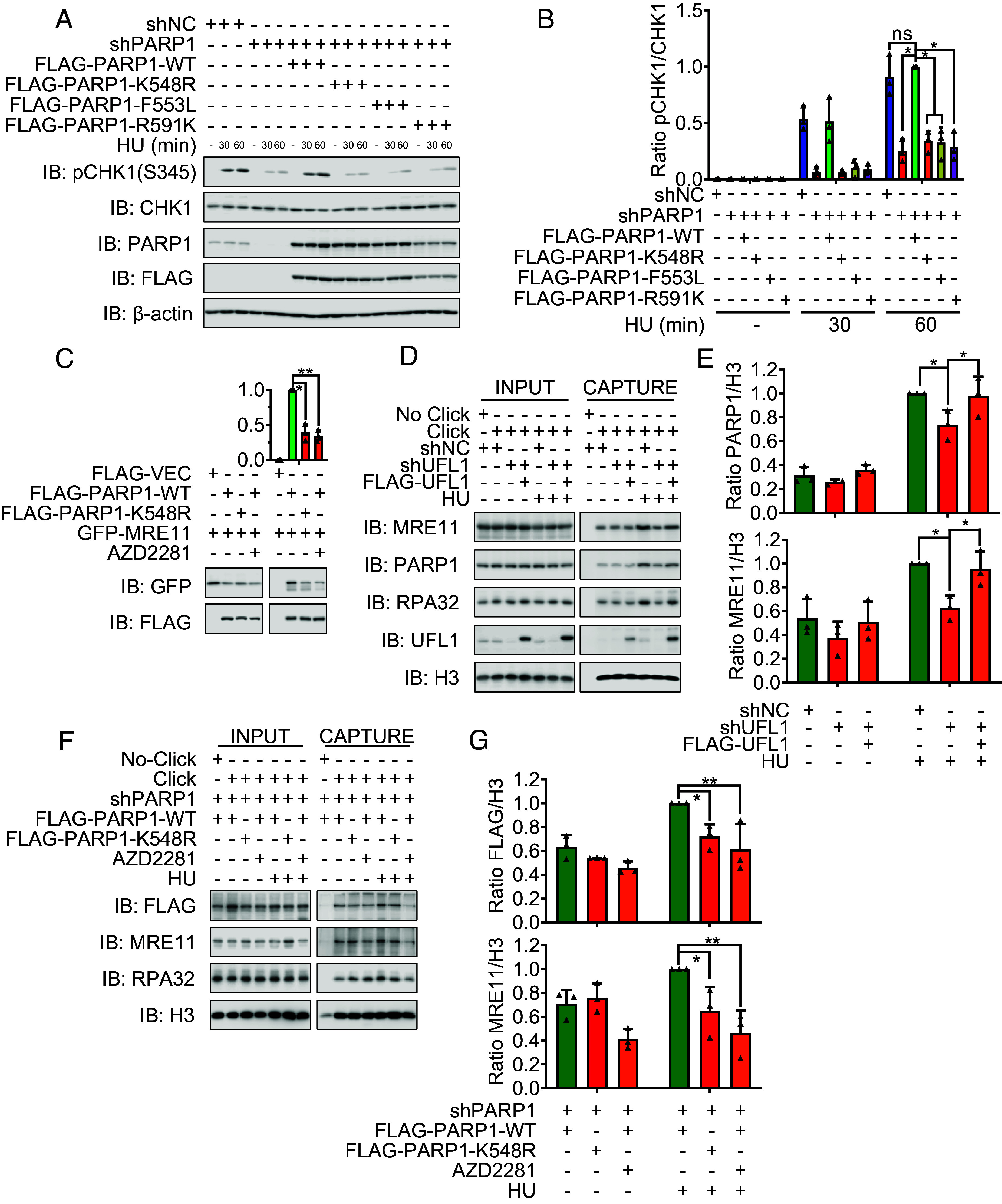
Defective PARP1 UFMylation impairs CHK1 activation and MRE11 recruitment to the stalled replication forks. (*A* and *B*) PAPR1 UFMylation deficiency compromised the activation of CHK1. (*A*) The stable HeLa cell lines of shNC (negative control), shPAPR1 (PARP1 depleted), and FLAG-PARP1 (shPARP1-resistant PARP1 cDNA reexpressed) WT and mutations (K548R, F553L, and R591K) treated with or without HU (2 mM) for the indicated times were harvested. The activation of CHK1 was analyzed by SDS-PAGE and WB (*A*) with indicated antibodies. (*B*) The respective quantifications for activation of CHK1 are shown, and a column plot with bar of the ratios of pCHK1 and CHK1 is shown. Two-way ANOVA (Tukey’s multiple comparisons test): ns = nonsignificant; *P < 0.05, **P < 0.01, N = 3. (*C*) PARP1 UFMylation regulates its interaction with MRE11. FLAG-PARP1 WT, K548R, or Vector, together with GFP-MRE11, were transfected into HEK293T cells, and the cells were lysed and subjected to FLAG affinity gel to immunoprecipitate FLAG-PARP1 and the immunoprecipitates were analyzed by SDS-PAGE and WB (*Bottom*) with indicated antibodies. AZD2281: PARP1 inhibitor which was added (10 μM, 1 h) before cell harvest. The respective quantifications for PARP1 UFMylation are shown, and a column plot with bar of the ratios of HA and FLAG (*Top*) is shown. Paired *t* test: *P < 0.05, **P < 0.01, N = 3. (*D* and *E*) UFL1 depletion reduces the relocalization of PARP1, MRE11, and RPA32 to stalled replication. The stable HeLa cell lines of shNC (negative control), shUFL1 (UFL1 depleted), and FLAG-UFL1 (shUFL1-resistant UFL1 cDNA reexpressed) with or without HU treatment (4 mM, 4 h) were lysed and subjected to iPOND assay to isolate replication forks and the isolated complexes were analyzed by SDS-PAGE and WB (*D*) with indicated antibodies. (*E*) The respective quantifications for PARP1 (*Top*) and MRE11 (*Bottom*) relocalization are shown, and a column plot with bar of the ratios of PARP1 (*Top*) or MRE11 (*Bottom*) to H3, respectively, is shown. Two-way ANOVA (Tukey’s multiple comparisons test): *P < 0.05, N = 3. (*F* and *G*) PARP1 UFMylation deficiency reduces the relocalization of PARP1, MRE11 and RPA32 to stalled replication. The stable HeLa cell lines of FLAG-PARP1 (shPARP1-resistant PARP1 cDNA reexpressed) WT and mutation (K548R) with or without HU treatment (4 mM, 4 h) were treated as (*D*). AZD2281: PARP1 inhibitor which was added (10 μM, 1 h) before cell harvest. (*G*) The respective quantifications for FLAG-PARP1 (*Top*) and MRE11 (*Bottom*) relocalization are shown, and a column plot with bar of the ratios of FLAG (*Top*) or MRE11 (*Bottom*) to H3, respectively, is shown. Two-way ANOVA (Tukey’s multiple comparisons test): ns = nonsignificant; *P < 0.05, N = 3.

It is known that PARP1 regulates CHK1 activation to promote S-phase checkpoint activation in response to replication stress (32, 33). The next logical step, therefore, was to examine the integrity of the S-phase checkpoint in the context of PARP1 UFMylation by analyzing CHK1 activation following HU treatment. We observed that PARP1 depletion led to decreased CHK1 activation, which was restored by reexpressing PARP1-WT, but not K548R-, F553L-, or R591K-mutant PARP1 in both HeLa and MGC803 cells (Fig. 4 *A* and *B* and *SI Appendix*, Fig. S4 *A* and *B*). Here, R591K was as a control of WGR domain mutation and inhibits its PARylation (31)**.** Thus, the PARP1 UFMylation is required for CHK1 activation in response to replication stress. To investigate whether CHK1 activation is due to a PARP1-CHK1 interaction, we performed an IP with endogenous PARP1 and detected CHK1 in UFL1-deficient cells. We found that UFL1 depletion did not attenuate the interaction between PARP1 and CHK1, indicating that while PARP1 UFMylation promotes CHK1 activation, this does not occur via the direct binding of the CHK1 protein (*SI Appendix*, Fig. S4*C*).

PARP1 recruits (through its PAR chains) MRE11 to stalled replication forks to process nascent DNA and promote forks restart (29, 34). Next, we tested whether PARP1 UFMylation regulates MRE11’s functions at stalled replication forks. We first performed an IP assay and found that the interaction between PARP1 and MRE11 is compromised by UFL1 depletion (*SI Appendix*, Fig. S4*C*). Similarly, we observed a reduced interaction of the PARP1-K548R mutant with MRE11 to a similar level to that seen in cells exposed to the PARP inhibitor AZD2281 (Fig. 4*C* and *SI Appendix*, Fig. S4*D*). To further confirm that PARP1 UFMylation promotes MRE11 recruitment to the replication fork under replication stress, we isolated replication forks by iPOND assay to detect the proteins at replication forks by antibody as indicated (Fig. 4 *D*–*G*). Here, we observed that PARP1, MRE11, and RPA32 protein levels were reduced at stalled replication forks derived from UFMylation-deficient cells (shUFL1) compared with control cells; this effect was rescued upon the reexpression of UFL1 (Fig. 4 *D* and *E*). We thus infer that UFMylation regulates PARP1-dependent MRE11 recruitment to stalled replication forks. Moreover, the PARP1-K548R mutation and AZD2281 treatment both resulted in reduced PARP1 recruitment to stalled replication forks and decreased MRE11 recruitment (Fig. 4 *F* and *G*). We thus conclude that PARP1 UFMylation controls PARP1 and MRE11 recruitment and their functions at stalled replication forks.

### Defective PARP1 UFMylation Impairs Stalled Replication Fork Degradation and Restart.

**Fig. 5.**
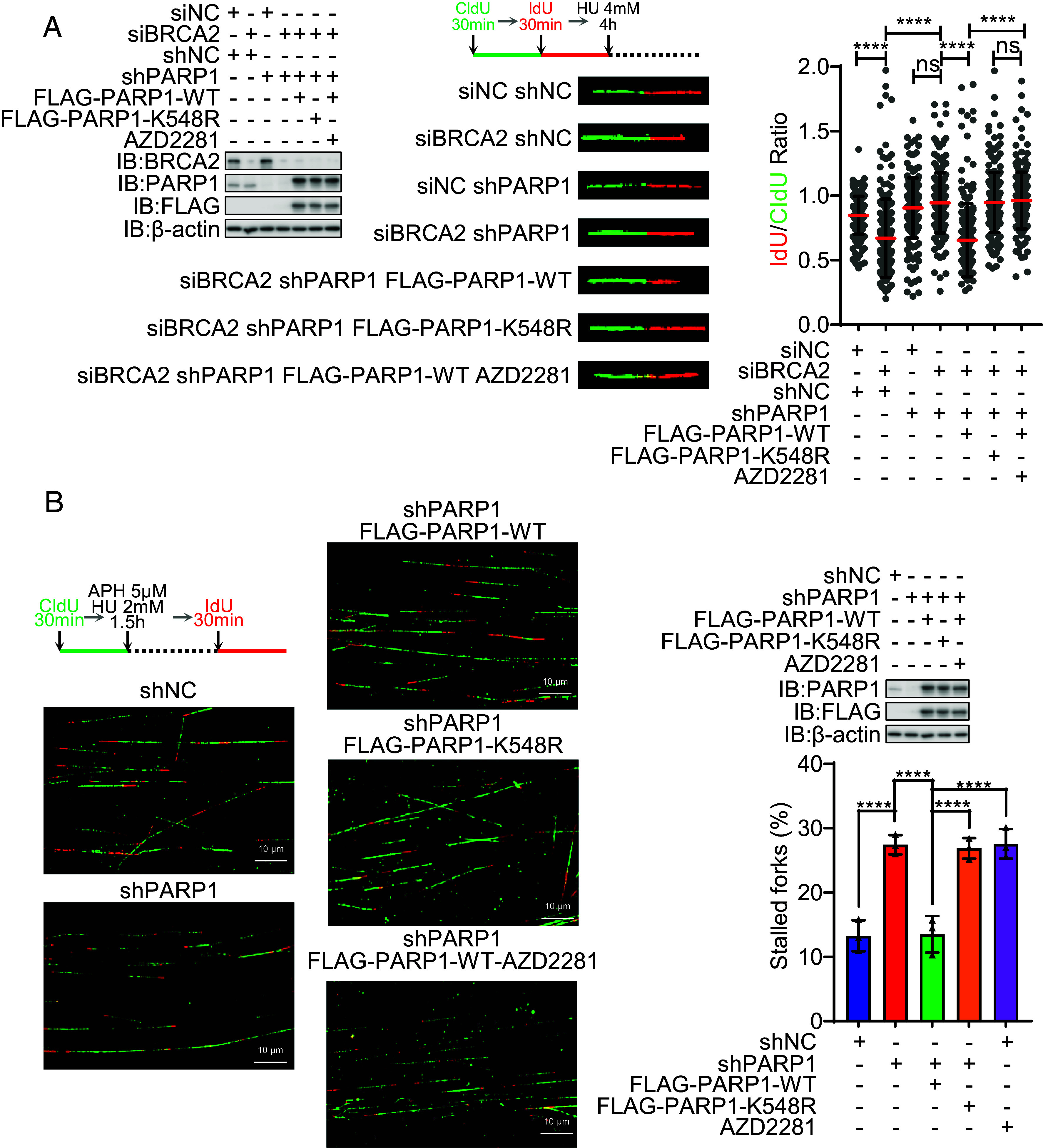
Defective PARP1 UFMylation impairs stalled replication fork degradation and restart. (*A*) PARP1 UFMylation deficiency compromised the degradation of stalled replication forks. The stable HeLa cell lines of shNC (negative control), shPAPR1 (PARP1 depleted), and FLAG-PARP1 (shPARP1-resistant PARP1 cDNA reexpressed) WT and mutation (K548R) were transfected with siBRCA2 and then labeled with CldU and then IdU, before HU treatment (4 mM, 4 h), and then, the DNA fiber assay was conducted to determine the degradation of stalled replication forks. At least 100 tracts were counted, and the experiment was repeated three times. Protein expression levels identified by WB (*Left*), representative images of CldU (green) and IdU (red) replication tracks (*Middle*), and a scatter plot of IdU/CldU tract length ratios (*Right*) for individual replication forks are shown. Unpaired *t* test (Mann–Whitney test): ns = nonsignificant; ****P < 0.0001. (*B*) PARP1 UFMylation deficiency compromised the restart of stalled replication forks. The stable HeLa cell lines of shNC (negative control), shPAPR1 (PARP1 depleted), and FLAG-PARP1 (shPARP1-resistant PARP1 cDNA reexpressed) WT and mutation (K548R) were labeled with CldU and then HU treatment (HU 2 mM for 1.5 h) and then labeled with IdU. The cells were harvested immediately, and the DNA fiber assay was conducted to determine the restart of stalled replication forks. At least 100 tracts were counted, and the experiment was repeated three times. Representative images of CldU (green) and IdU (red) replication tracks (*Left*), protein expression levels identified by WB (*Right Up*), and a column plot with bar of the ratios of CldU without IdU tracts (*Right Bottom*) for replication forks are shown. Paired *t* test: ****P < 0.0001. IB: immunoblot; WB: western blotting; IP: immunoprecipitation; HU: hydroxyurea; Dox: doxycycline. H3 and β-actin were used as loading controls.

MRE11 mediates nascent DNA resection, which is necessary to promote replication fork restart (35). As such, we next analyzed stalled replication fork stability and restart. Because nascent DNA resection by MRE11 is inhibited by BRCA1/2 (35), we needed to use a BRCA2-deficient background to detect obvious fork degradation (Fig. 5*A*). Like PARP1 depletion and AZD2281, we found that PARP1-K548R prevented nascent DNA resection, confirming that PARP1 UFMylation enhances its catalytic activity which promotes MRE11-mediated nascent DNA resection at stalled replication forks (Fig. 4 *F* and *G*). Consistently, PARP1 depletion, K548R mutation, and AZD2281 all largely compromised stalled replication fork restart (Fig. 5*B*).

### PARP1 UFMylation Promotes Genome Stability and Protects Mice from Genotoxic Stress.

**Fig. 6.**
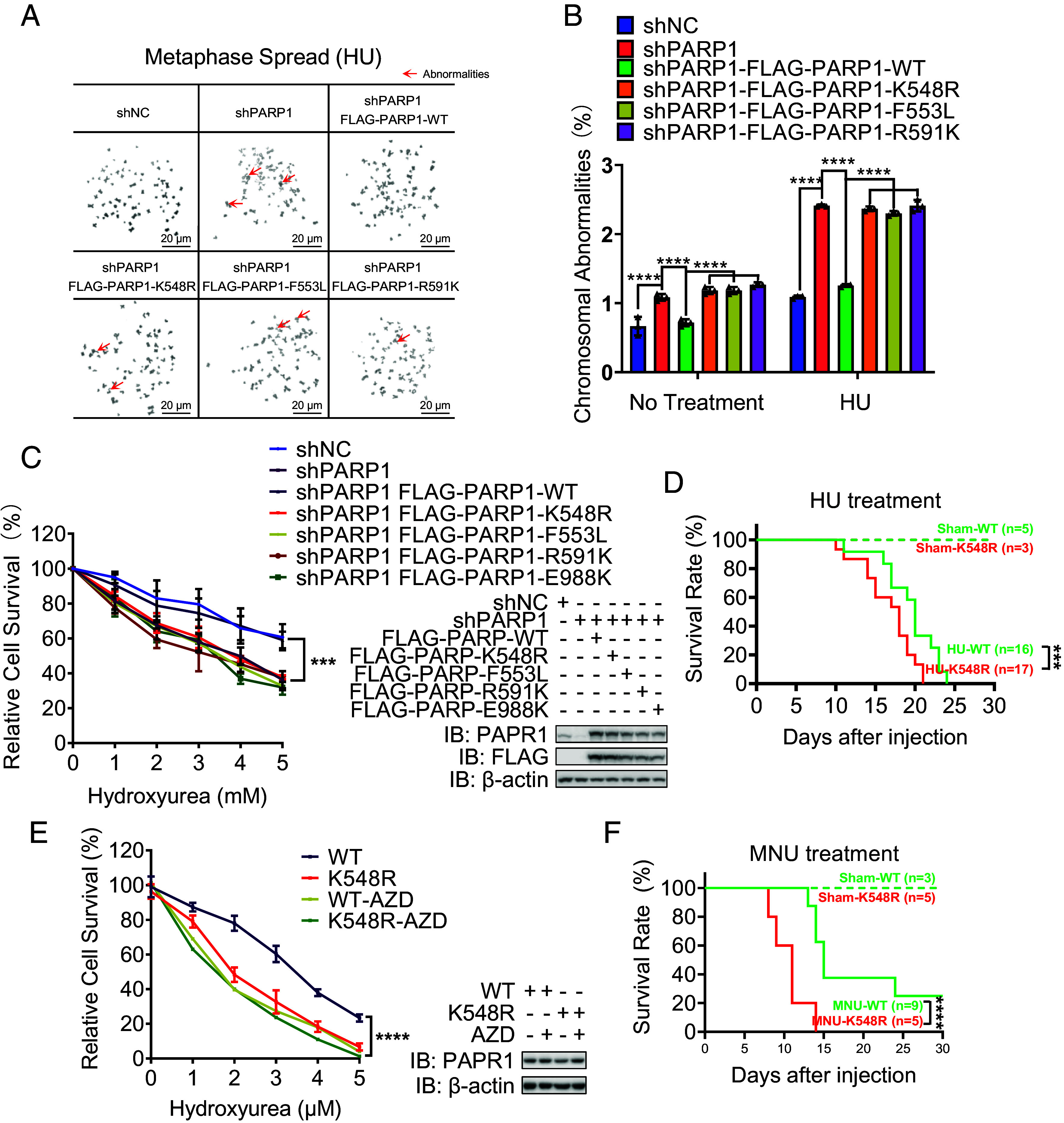
PARP1 UFMylation promotes genome stability and mice viability. (*A* and *B*) PARP1 UFMylation deficiency decreased genome stability. The stable HeLa cell lines of shNC (negative control), shPAPR1 (PARP1 depleted), and FLAG-PARP1 (shPARP1-resistant PARP1 cDNA reexpressed) WT and mutations (K548R, F553L, and R591K) were treated with HU (4 mM, 5 h) followed by nocodazole (200 ng/mL, 16 h) and then subjected to mitotic spread analysis. (*A*) Representative images of mitotic spreads; the red arrow indicates abnormal chromosome. (Scale bar: 20 μm.) (*B*) Bar chart of the % chromosomal abnormalities. Two-way ANOVA (Tukey’s multiple comparisons test): ****P < 0.0001. (*C*) PARP1 UFMylation deficiency sensitized cells to replication stress. HeLa cells stably expressing shNC, shPARP1, or shPARP1 with rescued PARP1-WT/K548R/F553L/R591K were treated with increasing concentrations of HU as indicated. The surviving colonies were analyzed after 14 d. The % surviving cells (*Left*) and the corresponding protein expression levels determined by WB (*Right*) are shown. Two-way ANOVA (Tukey’s multiple comparisons test): ***P < 0.001. (*D*) PARP1 UFMylation deficiency sensitized mice to replication stress. The survival of mice (age: 2 to 3 mo) with the indicated PARP1 genotypes (WT: PARP1^WT/WT^, K548: PARP1^K548R/K548R^) after receiving a single dose of HU (2,000 mg/kg of body weight) or solvent (sham) by intraperitoneal injection was shown. Log-rank (Mantel–Cox test) test: ***P < 0.001. (*E*) Cells from PAPR1^K548R/K548R^ mice are sensitive to HU treatment. PARP1^WT/WT^ (WT) and PARP1^K548R/K548R^ (K548R) iMEF cells were treated with increasing concentrations of HU as indicated. The surviving colonies were analyzed after 14 d. The % surviving cells (*Left*) and the corresponding protein expression levels determined by WB (*Right*) are shown. Two-way ANOVA (Tukey’s multiple comparisons test): ns=nonsignificant; **P < 0.01; ***P < 0.001. (*F*) PARP1 UFMylation deficiency sensitized mice to MNU. The survival of mice (age: 2 to 3 mo) with the indicated PARP1 genotypes (WT:PARP1^WT/WT^, K548: PARP1^K548R/K548R^) after receiving a single dose of HU (150 mg/kg of body weight) or solvent (sham) by intraperitoneal injection was shown. Log-rank (Mantel–Cox test) test: ****P < 0.0001. WB: western blotting; IB: immunoblot; HU: hydroxyurea; AZD: AZD2281, PARP1 inhibitor; β-actin were used as loading controls.

Defects in replication fork restart lead to replication fork collapse and ultimately induce genome instability (36). PARP1 enzymatic activity is essential for cells and mice in genotoxic response (37–39). To study the biological effect of PARP1 UFMylation in genotoxic stress response, we first performed a metaphase chromosome analysis to assess the level of chromosome aberrations after HU treatment. We found that the PARP1 K548R and F553L mutations all significantly increased the level of chromosome aberrations, especially with HU treatment (Fig. 6 *A* and *B* and *SI Appendix*, Fig. S5*A*), indicating that PARP1 UFMylation and its activity is important for maintaining genome stability.

Replication stress is responsible for genome instability and sensitivity of cells (3, 40). To understand the basis of genomic stability, we examined the influence of UFMylation on cellular sensitivity to replication poison. The survival rate of shRNA-mediated UFL1-depleted cells was decreased after HU treatment, and this effect was rescued upon the reexpression of UFL1 (*SI Appendix*, Fig. S5*B*). As expected, PARP1 depletion sensitized cells to HU treatment, which can be reversed by reexpression of PARP1 WT, but not its UFMylation mutants K548R or F553L, similar to catalytic mutants R591K and E988K, or by AZD2281 treatment (Fig. 6*C* and *SI Appendix*, Fig. S5*C*). These results indicate that PARP1 UFMylation ensures its catalytic activity, which is important for cell survival in response to replication stress.

Finally, we assessed the effects of PARP1 UFMylation during the replication stress response in mice. Consistent with the results from cells, after a single intraperitoneal (i.p.) injection of HU, there was significant increase of lethality in homo mutant mice compared to WT controls, with all homozygous PARP1^K548R/K548R^ mice died after 3 wk (Fig. 6*D*). This indicates that PARP1 K548R knock-in mice were more sensitive to HU compared to WT (Fig. 6*D*). Additionally, we isolated primary MEF cells from these mice and performed a colony formation assay. Similar to WT cells treated with PARP inhibitor AZD2281, PARP1-K548R knock-in cells were more sensitive to HU treatment compared with those from WT mice (Fig. 6*E*). We also detected these cells’ response to replication stress and found that similar to human cancer cells, CHK1 activation was impaired in PARP1-K548R knock-in MEF cells compared with that in cells from WT mice (*SI Appendix*, Fig. S5*D*). UFMylation-mediated PARylation activity of PARP1 is essential for cellular sensitivity. Therefore, we injected mice with MNU, an alkylating agent which can largely activate PARP1 (33), and strikingly found that the homozygous PARP1^K548R/K548R^ mice are hypersensitive to MNU treatment comparing with PARP1 WT mice (Fig. 6*F*). Taken together, we conclude that PARP1 UFMylation which is required for its full catalytic activity plays important roles in protecting cells and mice from genotoxic stress.

## Discussion

Replication stress plays a crucial role in regulating genome stability and thus the development of cancer and aging. Understanding the mechanisms underlying this process holds significant implications for disease prevention and therapy (3, 41). While UFMylation, as a novel ubiquitin-like modification, has been shown involved in various cellular processes, including ER homeostasis, ER-phage, DSB repair, tissue development, and tumorigenesis (22, 25–27), its role in the replication stress response was reported previously. The current study demonstrated that UFL1 is found in stalled replication forks and PARP1 is UFMylated, which participates in solving stalled replication forks. Deficiency of UFL1 and PARP1 UFMylation both compromise stalled replication fork degradation and restart and CHK1 activation.

As a form of PTMs, UFM1 covalently links to substrates to regulate their functions by altering protein structure, localization, or binding patterns. We identified the key replication fork responder PARP1 as a UFMylation substrate. Further experiments revealed that the major UFMylation site is K548, located within the WGR domain and close to the automodification (AM, residues 494 to 524) domain of PARP1. Many modifications within the PARP1 AM domain affect its activity; for example, K508 methylation and K482 SUMOlation promote PAR formation (42, 43), while K498, K506, and K518 acetylation blocks PARylation (44). The WGR domain is also important for PARP1 catalytic activity, as the R591K mutation within this domain abolishes PAR forming activity (31). Our in vivo and in vitro assays have verified that the UFMylation mutation at K548 of PARP1 impairs PARP1 catalytic activity. However, we have yet to mechanistically explore how a UFM moiety conjugated to Lys548 in the WGR domain increases PARP1 catalytic activity, and how the UFL1 is recruited to stalled replication forks and specifically recognizes K548 over other lysine residues in PARP1. It is also noted that the ubiquitination of PARP1 at K548 was reported by a previous ubiquitome analysis (45) and is shown in the UbiNet 2.0 database (https://awi.cuhk.edu.cn/~ubinet/index.php) (46). However, the biological significance of this ubiquitination remains unclear, and the potential cross talk between UFMylation and ubiquitination at K548 is the subject of future studies.

The recruitment and subsequent activation of PARP1 at stalled replication forks is required for recruiting MRE11 and XRCC1 to process replication and thereby for CHK1 activation (33, 34, 40). We found that CHK1 activation is more robustly affected by UFMylation under conditions of prolonged HU treatment (1 h in HeLa cells and 2 h in A549 cells). This indicates that UFMylation-facilitated CHK1 activation does not occur in the early response to DNA replication stress. In addition, we have previously reported that PAR binding to CHK1 promotes its activation in response to HU treatment (33). Under BRCA1/2 limitation, MRE11 resects the nascent DNA and promotes fork restart, thereby maintaining fork stability and preventing cell death (30, 47). We found that a PARP1 UFMylation deficiency compromises the resection of nascent DNA in BRCA2-deficient cells and delayed stalled replication forks restart in BRCA2-sufficient cells. Correlating with these observations in cells, PARP1 UFMylation mutant K548R renders cells a hypersensitivity to replication stress and high degree of genomic instability. Similarly, K548R mice are also sensitive to replication poison. Intriguingly, while there is mild hypersensitivity of K548R cells (colony formation assay) and mice in response to replication-specific poison HU, these mutant mice are very sensitive to MNU, compared to WT mice. This increased sensitivity can be attributed to MNU-induced base excision repair (BER) and PARP1 activation, then impedes the repair and restart of replication forks eventually leading to DNA single-stranded breaks (SSBs) and DSBs, and causing cellular senescence (48).

Replication fork reversal is essential for replication fork degradation (49). It has been reported that PARP1 activity is required for effective fork reversal (50) and stabilizes forks in their regressed state by restricting their restart by RECQ1 (51). As PARP1 UFMylation enhances its activity, we reason that PARP1 UFMylation might also affect replication fork reversal and thereby its degradation. This scenario can be further tested through electron microscopy to visualize the replication intermediates (51).

**Fig. 7.**
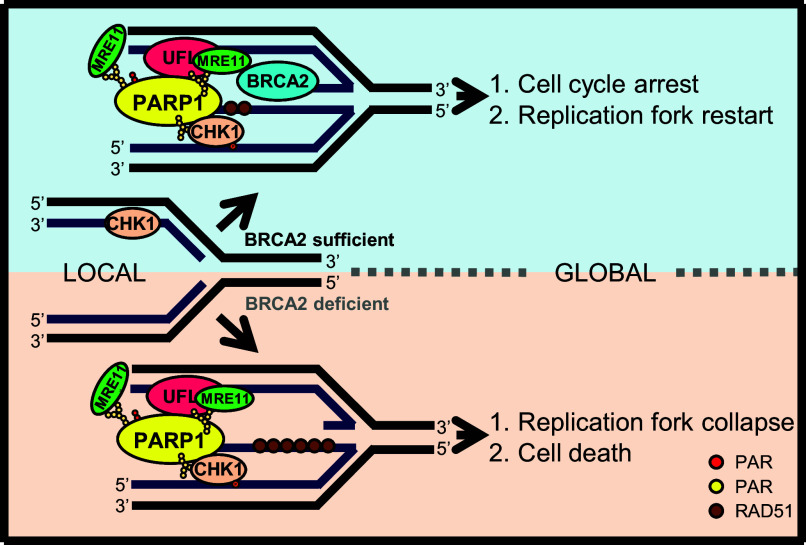
Working model of PARP1 UFMylation during the replication stress response. When cells are under replication stress, the replication fork reverses. PARP1 (yellow) and UFL1 (red) colocalize to the reversed replication forks, which leads to PARP1 UFMylation and enhances PARP1 activation (produce PAR). Activated PARP1 promotes the recruitment of MRE11 (green) for moderate and appropriate end resection with the supervision of BRCA1/2, which promotes fork restart. PARP1 also interacts with CHK1 (pink) to promote CHK1 activation and cell cycle arrest. When BRCA2 is deficient, PARP1 UFMylation will lead to MRE11-mediated overresection of replication forks, which leads to genome instability.

Based on our results, we propose a model by which PARP1 UFMylation mediates the replication stress response (Fig. 7). When a reversed replication fork occurs, PARP1 and UFL1 are recruited to the replication forks. Here, UFL1 mediates PARP1 UFMylation, enhancing PARP1 activity to produce PAR, which recruits MRE11 to nascent DNA for resection, a necessary step for stalled replication fork restart. Simultaneously, enhanced PARP1 activity promotes S-phase checkpoint activation halting cell cycle, ensuring enough time to process the stress. In BRCA1/2-deficient cells, without BRCA1/2 limitation, PARP1 UFMylation leads to excessive MRE11-mediated nascent DNA resection, and ultimately replication fork collapse and genome instability.

PARP1 is a multiple functional protein: It is activated by oxidative DNA damage and SSBs to recruit BER machinery (39, 48, 52), and helps to regulate DSBs, promoting the microhomology-mediated end joining at DSBs (14, 53, 54). In addition to the replication stress response, we found that UFMylation of PARP1 ensures its role in BER, as mutant mice are even more sensitive to MNU. Different from HU, MNU, an alkylating agent, can alkylate DNA to produce DNA adducts, which is repaired through BER machinery and PARP1 activation; therefore, low activity of PARP1 leads to hypersensitivity to MNU (33). PARP1 inhibitors induce cell death in BRCA1/2-deficient cells, a phenomenon known as synthetic lethality due to the decreased restart of the replication fork (29) and impaired homologous recombination (55). It is plausible that PARP1 UFMylation may play a role in this synthetic lethality. Targeting UFMylation machinery through both replication fork stability and BER may present an alternative and promising approach for synthetic killing cancer cells by modulating PARP1 activity.

## Materials and Methods

In the present study, iPOND was used for isolation of proteins on the replication fork, DNA fiber analysis was employed to determine DNA replication and replication fork status, mitotic spread analysis was used to determine chromosome stability, homologous recombination-based gene-targeting was employed to generate Parp1(K548R) knock-in mice. Details of the experiments, e.g., molecular cloning and transfection, biochemical and cellular assays, cell culture, generation and characterization of knock-in mice, and statistical analysis, are described in *SI Appendix*, *Materials and Methods*.

## Supplementary Material

Appendix 01 (PDF)

## Data Availability

All study data are included in the article and/or *SI Appendix*.
