# Supplementary material for: PARP1 UFMylation ensures the stability of stalled replication forks
Source: Proc Natl Acad Sci U S A. 2024 Apr 24;121(18):e2322520121. doi: 10.1073/pnas.2322520121 (PMC11066985; doi:10.1073/pnas.2322520121)
Supplement: Supplementary file 1 — Appendix 01 (PDF) [file pnas.2322520121.sapp.pdf]

Supplementary Materials and Methods

Cell culture, cDNA synthesis, plasmid construction, and drugs

Human HeLa, U2OS, MGC803, A549, HEK293T, and mice iMEF cells were cultured at 37˚C in DMEM medium supplemented with 10% fetal bovine serum and 1% penicillin/streptomycin (Hyclone) in a humidified incubator with 5% CO_2.

RNA isolation and reverse transcription (RT) was carried out as previously described (1). RNA was isolated from cells using RNase Mini Kit (74104, QIAGEN) according to the manual. First strand cDNA was then synthesized from 0.5 μg of RNA using HiScript® II 1st Strand cDNA Synthesis Kit according to the manual (R212-01/02, Vazyme).

PARP1 and UFL1 cDNAs were sub-cloned into a lenti-blast vector (#V005060, Novo Bio). PARP1 cDNA was cloned into an EGFP-C1 expression vector (#V012024, Novo Bio). UFM1 and UfSP2 cDNAs were cloned into a pcDNA3.0-HA vector (#V012404, Novo Bio). UBA5, UFC1, UFL1 and UfBP1 cDNAs were cloned into a pcDNA3.1-MYC-HIS vector (#V011332, Novo Bio). Bacteria expressing HIS-tagged UBA5, UFC1, UFL1, UFM1, UfSP2 and HIS-tagged PARP1 plasmid were cloned into a pET28a vector (69864, Novagen). shRNAs targeting endogenous PARP1, or UFL1 were generated using pLKO.1 vector (#21915, Addgene). Mutations in PARP1, UFM1, UfSP2, and shRNA target site-resistant mutations in UFL1 were generated using a Mut Express II Fast Mutagenesis Kit V2 (C214, Vazyme).

HU (H8627-5G), Nocodazole (M1404), IdU (#I7125), and CldU (#C6891) were purchased from Sigma-Aldrich. Puromycin (S7417), Blasticidin S HCl (S7419), and

Doxcycline (S5159) were purchased from Selleck. EdU (E10187) was purchased from Invitrogen.

Because high concentration and long-treatment of HU lead to replication fork collapse and subsequent double-strand breaks, the amount and duration of HU treatments vary dependent on the experiment aims. For isolation of proteins on nascent DNA (iPOND) and degradation of stalled replication forks, we treated cell with 4 mM for 4 h to completely block the replication fork and promote the recruitment of end resection factors (2, 3). For mitotic spread assays, we treated cell with 4 mM HU for 5 h to completely block the replication fork and induce genomic instability (4). For the restart of stalled replication forks, we treated cell with 2 mM HU for 1.5 h to block replication forks (5). For the CHK1 activation we treated cell with 2 mM HU for the indicated time to induce S-phase checkpoint (6).

siRNA/shRNA

Endogenous PARP1 expression was knocked down using siRNA/shRNA: 5'-TGGAAAGATGTTAAGCATTTA-3' (7). Endogenous BRCA2 expression was knocked down using the siRNA: 5'-GAAGAATGCAGGTTTAATA-3' (8). Endogenous UFL1 expression was knocked down using the siRNA: 5'-CAGGGAGATTAUCCCTTGA-3' (9), and the shRNA: 5'-GTTCCAACATCGACAAGCA-3' (9). siRNAs were transfected into cells using lipofectamine RNAiMAX reagent (Invitrogen), according to the manufacturer’s manual, and cells were analyzed 48 h after the siRNA transfection.

Generation of stable cell lines

We used three-plasmid expression system (psPAX2 5μg, pMD2.G 2.5μg, and relevant vector construct 2.5μg) to generate lentivirus-based vectors by transient transfection of 293T cells. Endogenous PARP1 (shPARP1) or UFL1 (shUFL1) knockdown in HeLa, MGC803, or A549 cells was achieved by shRNA lentiviral infection and puromycin selection (2 μg/ml) for 2 weeks to establish the stable cell lines (9). The stably silenced cells were reconstituted with a shRNA-resistant form of FLAG-UFL1 or FLAG-PARP1-WT/K548R/F553L/R591K/E98K through lentiviral infection and selected on blasticidin (10 μg/ml) for 2 weeks to establish the stable cell lines (9).

Immunoblotting, immunoprecipitation, pull-down assays and immunofluorescence

First antibodies: anti-HA (A190-208A), anti-MYC (A190-205A), anti-UFL1 (A303-455A, A303-456A), anti-PARP1 (A301-375A), anti-CHK1 (A300-298A), anti-BRCA2 (A303-434A), anti-RPA32 (A300-244A), and anti-pRPA32 (S33) (A300-246A) were purchased from Bethyl. Anti-PAR (ab14459), anti-BrdU (ab6326), anti-H3 (ab1791), and anti-MRE11 (ab214) was purchased from Abcam. Anti-FLAG (F1804) and anti-β-actin (A5441) were purchased from Sigma-Aldrich. Anti-GFP (sc-9996) and anti-UfSP2 (sc-376084) was purchased from Santa Cruz. Anti-HIS (D291-3) was purchased from MBL. Anti-pCHK1 (S345) (#2341) was purchased from Cell Signaling Technology. Anti-PAR (4335-MC-100-AC) was purchased from Trevigen. Anti-BrdU (347580) was purchased from BD.

Second antibodies: anti-Mouse IgG (H+L) HRP (115-035-116), anti-Mouse IgG (L) HRP (115-035-174), anti-Rabbit FITC (111-095-144), and anti-Rat Alexa Fluor 488

(712-546-150) were purchased from Jackson, anti-Mouse Alexa Fluor 594 (A21203) was purchased from Life Technologies.

Immunoblotting (IB) and immunoprecipitation (IP) were performed as previously described (9). Briefly, the harvested cells were lysed in RIPA Buffer at 4°C for 30 min. The lysates were centrifuged at high speed and then immunoprecipitated with the appropriate antibodies. IP in denaturing conditions was performed as previously described (9). Briefly, the harvested cells were lysed in SDS Lysis Buffer (150 M Tris, pH 8.0, 5% SDS, 30% glycerol) at 100°C for 10 min before digestion with benzonuclease (E1014, Sigma-Aldrich) at 37°C for 30 min. The lysates were centrifuged at high speed and then diluted 20 times with RIPA buffer and the FLAG tagged proteins were subjected to immunoprecipitation with FLAG affinity gel (Bimake, B23102). The precipitates were then analyzed by SDS-PAGE and Western Blot and the proteins on the membranes were detected by indicated antibodies.

Pull-down assays were performed as previously described (9). Briefly, bacterially purified HIS-FLAG-PARP1 and HIS-UFL1/UfSP2 immobilized on FLAG affinity gel were incubated for 4 h at 4°C. The precipitates were then analyzed by SDS-PAGE and Western Blot. Following SDS-PAGE and transfer to PVDF membrane, the membrane was coated with appropriate first antibodies were dissolved in blocking solution for overnight at 4°C. First antibodies were detected by indicated second antibodies by incubating at room temperature for 1 h.

Immunofluorescence of PAR and UFL1 was performed as previously described (10). Briefly, HeLa cells were fixed with 4% paraformaldehyde (PFA) at 4°C for 10 min

before incubation in cold methanol at -20°C for 20 min. A PAR mouse monoclonal antibody and rabbit UFL1 antibody (1:500) were applied in blocking solution for overnight at 4°C, and then fluoresces labeled secondary antibodies (1:250) were applied in blocking solution for 30 min at 37°C. Images were captured under a DragonFly confocal imaging system (Andor).

Protein purification

Protein expression and purification were performed as previously described (9). FLAG-tagged PARP1-WT/K548R/F553L/R591K/E988K proteins were generated from HeLa cells, purified using FLAG affinity gel, and eluted using a FLAG peptide. HIS-UBA5, HIS-UFC1, HIS-UFL1, HIS-HA-UFM1-ΔC2, and HIS-FLAG-PARP1-WT/K548R proteins were expressed in BL21 cells and purified using Ni-NTA agarose (Qiagen).

***In vitro* UFMylation assay and *in vitro* PARylation assay**

An *in vitro* UFMylation assay was performed as previously described (9). Briefly, purified HIS-UBA5 (0.1 μM), HIS-UFC1 (0.1 μM), HIS-UFL1 (0.1 μM), HIS-HA-UFM1-ΔC2 (0.1 μM) and HIS-FLAG-PARP1 (0.1 μM) were mixed in reaction buffer [0.05% bovine serum albumin, 50 mM 4-(2-hydroxyethyl)-1-piperazineethanesulfonic acid (HEPES), pH 7.5, 10 mM $MgCl_2$] containing 5 mM γ-adenosine triphosphate (ATP) and incubated at 30°C for 90 min. The mixtures were boiled in SDS lysis buffer, and then diluted 20 times with RIPA and immunoprecipitated with FLAG affinity gel.

An *in vitro* PARylation assay was performed as previously described (11, 12). Briefly, PARP1 was subjected to *in vitro* PARylation at 30°C for 30 min in the reaction buffer (50 mM Tris-HCl pH 8, 25 mM $MgCl_2$, 50 mM NaCl) supplemented with 200

μM NAD^+. The reaction was stopped by adding SDS sample buffer.

For *in vitro* PARylation assay after *in vitro* UFMylation. First PARP1 was subjected to UFMylation as described above. After UFMylation, 200 μM NAD^+ was added to the reaction complex and subjected to *in vitro* PARylation at 30°C for 30 min. The reaction was stopped by adding SDS sample buffer.

Mitotic spread analysis

Mitotic spread assays were performed as previously described (13). Briefly, HeLa cells were depleted of PARP1 and then reconstituted with FLAG-PARP1-WT/K548R/F553L/R591K before treatment with 4 mM HU for 5 h. HU was removed by washing three times with PBS, and the cells were then released into fresh medium containing 200 ng/ml nocodazole for 16 h before harvesting. Chromosome spreads were prepared after treating the cells with a hypotonic solution containing 56 mM KCl, and then fixed in methanol/acetic acid (volume ratio of 3:1) and stained with Giemsa. The images were captured under a DragonFly confocal imaging system (Andor). For each experiment, more than 400 mitotic chromosomes were randomly selected and analyzed (9).

DNA fiber analysis

A DNA fiber analysis was performed as previously described (14, 15). Briefly, cells were labeled with 30 μM CldU for 30 min, washed with PBS three times, and then labeled with 250 μM IdU for 30 min. Cells were then lysed in lysis buffer (0.5% SDS, 50 mM EDTA, 200 mM Tris-HCl, pH7.5), and DNA fibers stretched onto glass slides. The fibers were denatured with 2.5 N HCl at 37°C for 1 h, washed with PBS, and

blocked with 2% BSA in PBS containing 0.1% Tween-20 at 37°C for 30 min. The newly replicated CldU and IdU tracts were revealed with anti-BrdU antibodies recognizing CldU (rat, ab6326) and IdU (mouse, 347580), respectively, and secondary antibodies donkey anti-Mouse Alexa Fluor 594 and donkey anti-Rat Alexa Fluor 488 were applied in blocking solution for 30 min at 37°C. Images were captured under a DragonFly confocal imaging system (Andor), and statistical analysis was carried out using GraphPad Prism.

iPOND analysis

HeLa cells were labelled with 10 μM EdU, and treated with or without 4 mM HU for 4 h, as indicated (2, 3). The cells were then crosslinked with 1% formaldehyde for 10 min at room temperature, quenched with 0.125 M glycine, and washed with PBS. For the conjugation of EdU with biotin azide, the cells were permeabilized with 0.25% Triton X-100 in PBS buffer, and incubated in click reaction buffer (10 mM sodium-L-ascorbate, 20 μM biotin azide, 2 mM $CuSO_4$) for 2 h at room temperature. Subsequently, the cells were re-suspended in lysis buffer (50 mM Tris-HCl, pH 8.0), 1% SDS, protease inhibitor cocktail], and chromatin was solubilized by sonication at 4°C for 20 min. After centrifugation, supernatants were incubated for 1 h with Dynabeads M-280 Streptavidin (11206D, Invitrogen). The beads were then washed, and the captured proteins were eluted by boiling in SDS sample buffer for 20 min at 95°C.

Colony formation assay

The colony formation assay was performed as previously described (9). Briefly, 300 HeLa cells were seeded in 6-well plates with 2 ml complete medium (DMEM with 10%

Fetal Bovine Serum, 1% penicillin/streptomycin) for 24 h and then treated with 0, 1, 2, 3, 4, 5 mM HU for 4 h, and then changed to complete medium incubating for 2 weeks. The cell clones were counted and the sensitivities were calculated.

Generation of PARP1$^{K548R/K548R}$ mice

A gene-targeting vector containing the point mutation in PARP1 exon 12 was introduced into C57BL/6N embryonic stem (ES) cells by electroporation (16) **(Fig. S6A)**. Southern blot (SB) analysis of selected ES clones confirmed the presence of the targeted (Tg) and knock-in (K548R) allele mutation at the *NotI* locus, both before and after transfection with Cre-recombinase. To validate the Tg allele, genomic DNA was digested with either KpnI or BclI, followed by hybridization using a Neo probe. The Neo probe could identify the following DNA fragment from the Tg allele in the SB analysis: 13.73 kb (with KpnI digestion) and ~6.90 kb (with BclI digestion). For further validation, the genomic DNA was digested with either Bsu36I or SspI and hybridized using a 5'arm or 3'arm probe. The 5'arm or 3'arm probe could detect the following DNA fragment from the Tg allele in the SB analysis: 7.32 kb-WT, 9.15 kb-MT (with Bsu36I digestion) and 10.35 kb-WT, 8.98 kb-MT (with SspI digestion) **(Fig. S6C)**. The heterozygous PARP1 K548R (PARP1$^{+/K548R}$) ES clones were injected into blastocysts to generate chimeras which were subsequently crossed with C57BL/6 mice to establish the PARP1$^{K548R/K548R}$ founder lines. Genotyping of the animals was performed by PCR using the following primers: Forward (TCCTACTAGGCTATTCTCTTAGCCA) and Reverse (CACTGAAACTATCGCTATCATCCCAA) **(Fig. S6D)**. All sequences are given from 5' to 3' orientation.

Genotoxic treatment of mice

The required amount of HU was freshly dissolved in 0.9% (w/v) NaCl solution (pH < 5) and sterile-filtered prior to use. The animal body weights were measured, and the injection volume was calculated accordingly. The HU solution or solvent was administered intraperitoneally into mice aged 2–3 months of age.

Statistical analysis

GraphPad Prism 9 was used for statistical analyses. First, the data set were subjected to Shapiro-Wilk test to check for the normal distribution (p-value>0.05). Then, a Student's t test was used to determine the statistical significance between two groups. If the data set did not pass the Shapiro-Wilk test (p-value<0.05), the statistical significance was determined by Mann Whitney U test. Two-way-ANOVA was used for the comparison of more than two groups. A $p<0.05$ was considered to indicate statistical significance.

REFERENCE

1. Z.-W. Zhou *et al.*, An essential function for the ATR-activation-domain (AAD) of TopBP1 in mouse development and cellular senescence. *PLoS genetics* **9**, e1003702 (2013).
2. X. Ding *et al.*, Synthetic viability by BRCA2 and PARP1/ARTD1 deficiencies. *Nature communications* **7**, 1-12 (2016).
3. B. M. Sirbu, F. B. Couch, D. Cortez, Monitoring the spatiotemporal dynamics of proteins at replication forks and in assembled chromatin using isolation of proteins on nascent DNA. *Nature protocols* **7**, 594-605 (2012).
4. S. Mijic *et al.*, Replication fork reversal triggers fork degradation in BRCA2-defective cells. *Nature communications* **8**, 859 (2017).
5. S. Ying *et al.*, DNA-PKcs and PARP1 bind to unresected stalled DNA replication forks where they recruit XRCC1 to mediate repair. *Cancer research* **76**, 1078-1088 (2016).
6. W. Min *et al.*, Poly(ADP-ribose) binding to Chk1 at stalled replication forks is required for S-phase checkpoint activation. *Nature Communications* **4** (2013).
7. Y. Du *et al.*, Blocking c-Met–mediated PARP1 phosphorylation enhances anti-tumor effects of PARP inhibitors. *Nature medicine* **22**, 194-201 (2016).
8. F. Zhang, J. Shi, C. Bian, X. Yu, Poly (ADP-ribose) mediates the BRCA2-dependent early DNA damage response. *Cell reports* **13**, 678-689 (2015).

9. Z. Wang *et al.*, MRE11 UFMylation promotes ATM activation. *Nucleic acids research* **47**, 4124-4135 (2019).
10. L. Rank *et al.*, Analyzing structure–function relationships of artificial and cancer-associated PARP1 variants by reconstituting TALEN-generated HeLa PARP1 knock-out cells. *Nucleic acids research* **44**, 10386-10405 (2016).
11. V. Rolli, M. O'Farrell, J. Ménissier-de Murcia, G. de Murcia, Random mutagenesis of the poly (ADP-ribose) polymerase catalytic domain reveals amino acids involved in polymer branching. *Biochemistry* **36**, 12147-12154 (1997).
12. G. M. Shah *et al.*, Approaches to detect PARP-1 activation in vivo, in situ, and in vitro. *Poly (ADP-ribose) Polymerase: Methods and Protocols*, 3-34 (2011).
13. V. Bačovský, R. Hobza, B. Vyskot, Technical review: cytogenetic tools for studying mitotic chromosomes. *Plant Chromatin Dynamics: Methods and Protocols*, 509-535 (2018).
14. J. Nieminuszczy, R. A. Schwab, W. Niedzwiedz, The DNA fibre technique–tracking helicases at work. *Methods* **108**, 92-98 (2016).
15. A. Quinet, D. Lemaçon, A. Vindigni, Replication fork reversal: players and guardians. *Molecular cell* **68**, 830-833 (2017).
16. H. Schuhwerk *et al.*, Kinetics of poly (ADP-ribosyl) ation, but not PARP1 itself, determines the cell fate in response to DNA damage in vitro and in vivo. *Nucleic acids research* (2017).

Supplementary Figures

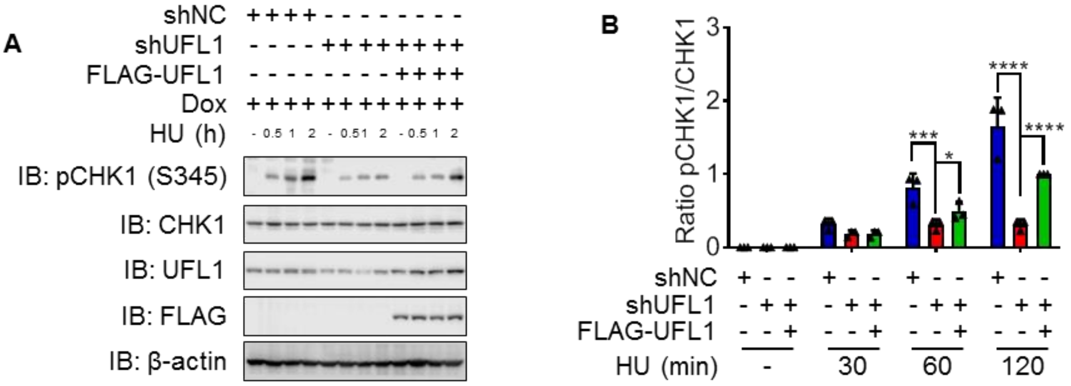

Fig. S1. Defective UFMylation compromises stalled replication fork stability and restart

(A) UFL1 depletion compromised the activation of CHK1. The stable A549 cell lines of shNC (negative control), shUFL1 (UFL1 depleted) and FLAG-UFL1 (shUFL1-resistant UFL1 cDNA re-expressed) treated with or without HU (2 mM) for the indicated times were harvested. The activation of CHK1 was analyzed by SDS-PAGE and WB with indicated antibodies.

(B) The respective quantifications for activation of CHK1 are shown, and column plot with bar of the ratios of pCHK1 and CHK1. Two-way ANOVA (Tukey's multiple comparisons test): *$p<0.05$, ***$p<0.001$, ****$p<0.0001$, N=3.

IB: Immunoblot; WB: Western blotting; HU: Hydroxyurea; Dox: Doxycycline. β-actin was used as loading control.

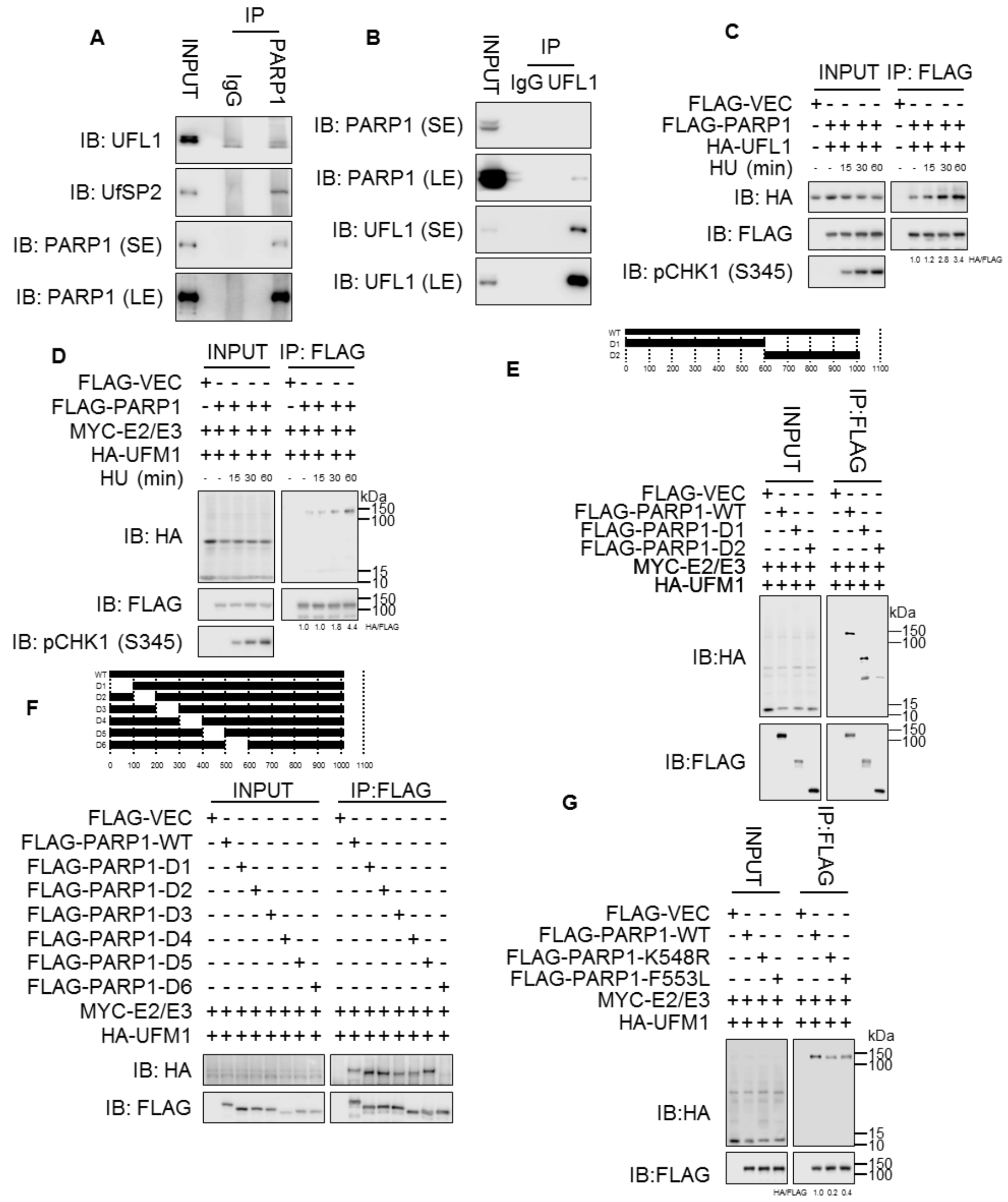

Fig. S2. PARP1 is UFMylated on K548

(A, B) PARP1 interacts with UFL1 and UfSP2. IP-WB of HEK293T cells with the indicated antibodies. **(C)** PARP1 dynamically interacted with UFL1 responding to HU treatment *in vivo*. FLAG-PARP1 or Vector, together with HA-UFL1, were transfected into HEK293T cells, which were HU treated (2 mM for indicated times) and lysed. The lysates were subjected to FLAG affinity gel to immunoprecipitate FLAG-PARP1 and the immunoprecipitates were analyzed by SDS-PAGE and WB with indicated

antibodies.

(D) PARP1 UFMylation dynamically responds to HU treatment. FLAG-PARP1 or Vector and HA-UFM1, together with MYC-UFC1 (E2) and MYC-UFL1 (E3), were transfected into HEK293T cells, which were HU treated (2 mM for indicated times) and subjected to denatured immunoprecipitation. The UFMylation levels of PAPR1 were analyzed by SDS-PAGE and WB with indicated antibodies.

(E, F) Identification of PARP1 UFMylation domain. FLAG-VEC/PARP1-WT, and FLAG-PARP1 deletion mutations, together with HA-UFM1, MYC-UFC1 (E2) and MYC-UFL1 (E3), were transfected into HEK293T cells, and the cells were subjected to denatured immunoprecipitation. The UFMylation levels of PAPR1 were analyzed by SDS-PAGE and WB with indicated antibodies. The upper panel shows PARP1 deletion mutations D1/D2/D3/D4/D5/D6.

(G) The K548R and F553L mutations of PARP1 decreased its UFMylation. FLAG-PARP1 WT, or K548R, or F553L, or Vector, together with HA-UFM1, MYC-UFC1 (E2) and MYC-UFL1 (E3), were transfected into HEK293T cells, and the cells were subjected to denatured immunoprecipitation. The UFMylation levels of PAPR1 were analyzed by SDS-PAGE and WB with indicated antibodies.

IB: Immunoblot; WB: Western blotting; IP: Immunoprecipitation.

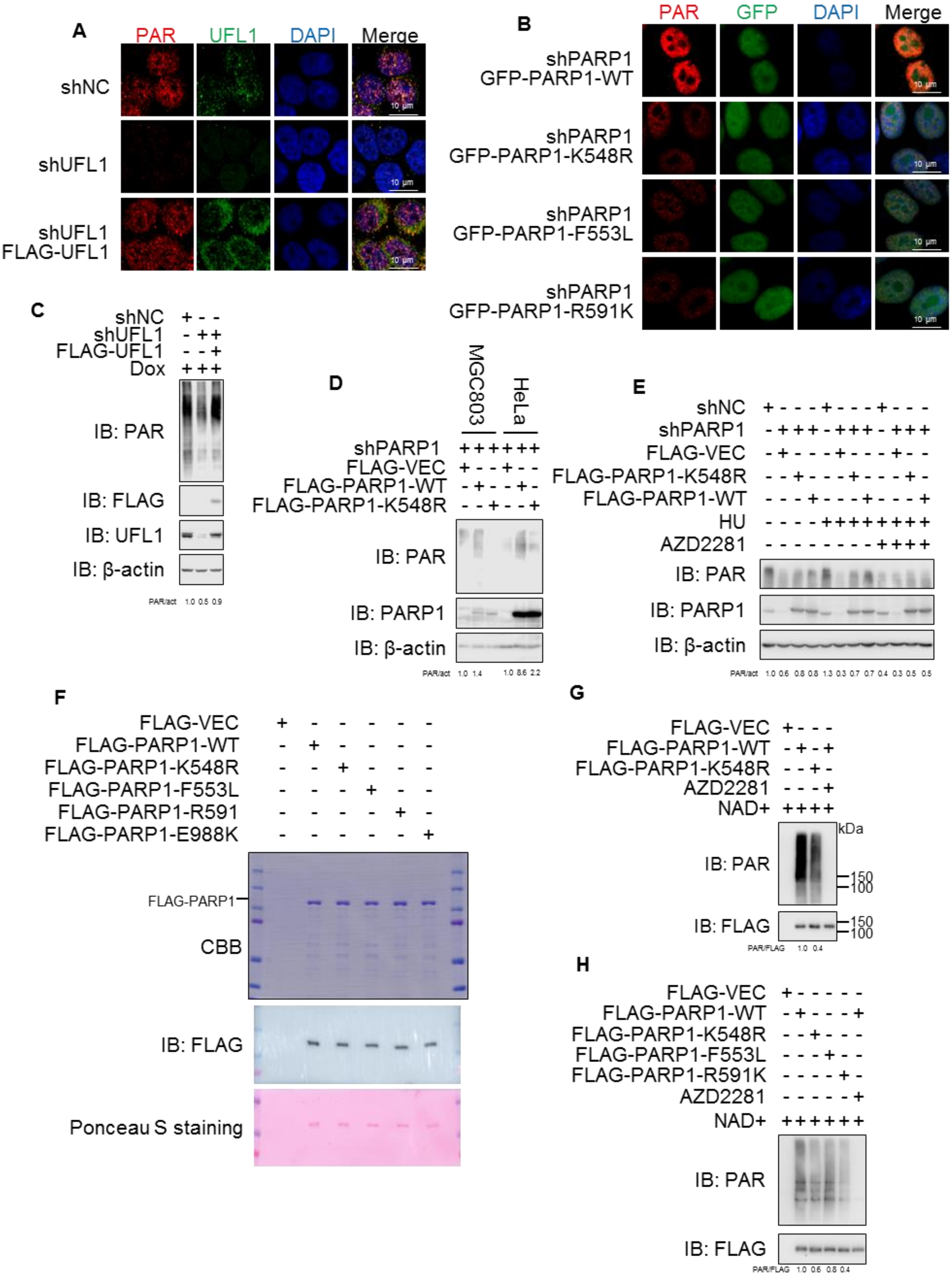

Fig. S3. PARP1 UFMylation promotes its PARylation activity

(A) UFL1 depletion decreased the signal of PAR (Zoom in of Fig. 3B). The stable HeLa cell lines of shNC (negative control), shUFL1 (UFL1 depleted) and FLAG-UFL1 (shUFL1-resistant UFL1 cDNA re-expressed) were seeded on cover slips and the PAR and UFL1 were detected with IF.

(B) PARP1 UFMylation and WGR domain are important for PAR formation (Zoom in of Fig. 3D). The stable HeLa cell lines of shPARP1 (PARP1 depleted) was transfected with GFP-PARP1 WT, K548R, F553L or R591K, and the PAR was detected with IF after 24 h transfection.

(C) UFL1 depletion decreased the signal of PAR. The stable HeLa cell lines of shNC (negative control), shUFL1 (UFL1 depleted) and FLAG-UFL1 (shUFL1-resistant UFL1 cDNA re-expressed) were harvested. The PAR signal was analyzed by SDS-PAGE and WB with indicated antibodies.

(D, E) PARP1 UFMylation and WGR domain are important for PAR formation. The stable MGC803 (D) or HeLa (D, E) cell lines of shNC (negative control), shPAPR1 (PARP1 depleted) and FLAG-PARP1 (shPARP1-resistant PARP1 cDNA re-expressed) WT and K548R mutation were harvested. The PAR signal was analyzed by SDS-PAGE and WB with indicated antibodies. (E) HeLa cells were treated with or without AZD2281 (10 μM, 1h) before treated with or without HU (2 mM, 1h) before harvest.

(F) Recombinant FLAG-tagged protein purification. Proteins were purified from FLAG-PARP1-WT/K548R/F553L/R591K overexpression HeLa cell lines using FLAG antibody and then eluted using FLAG peptide. The protein purification result was analyzed by SDS-PAGE and Coomassie blue staining or WB with indicated antibodies.

(G, H) PARP1 UFMylation deficiency impaired the PAR formation. The *in vitro* PARylation assay was conducted with incubating HeLa cell derived FLAG-PARP1 WT, or mutations (K548R, F553L, R591K, E988K), or Vector with NAD^+ at 30°C for 30 min. The reaction was terminated by adding SDS sample buffer and the samples were subjected to SDS-PAGE followed by immunoblotting with the indicated antibodies.

IF: Immunofluorescence; IP: Immunoprecipitation; IB: Immunoblot; AZD2281: PARP1 inhibitor.

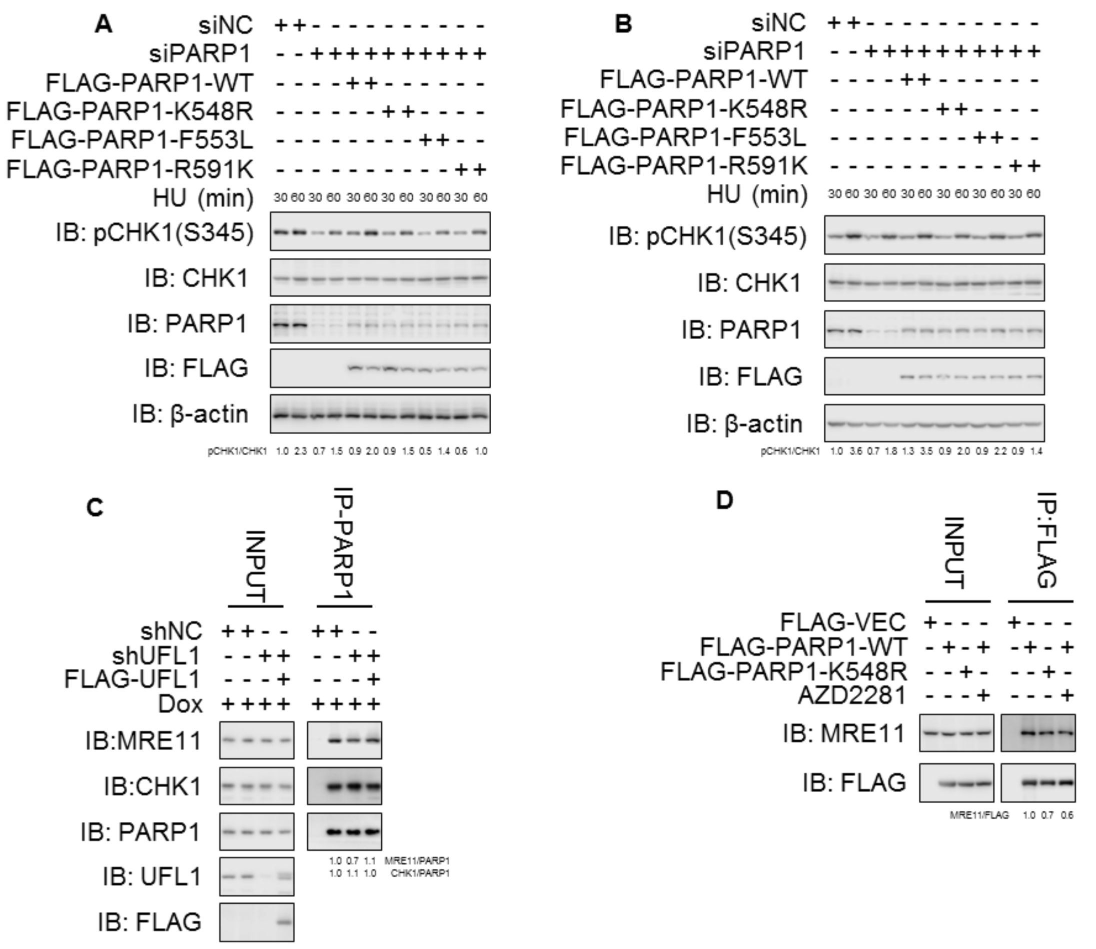

Fig. S4. Defective PARP1 UFMylation impairs CHK1 activation, stalled replication fork degradation and restart

(A, B) PAPR1 UFMylation deficiency compromised the activation of CHK1. The HeLa (A) or MGC803 (B) cells transfected with siNC (negative control), siPAPR1 (PARP1 depleted) and FLAG-PARP1 (shPARP1-resistant PARP1 cDNA re-expressed) WT and mutations (K548R, F553L, R591K) were treated with or without HU (2 mM) for the indicated times and were harvested. The activation of CHK1 was analyzed by SDS-PAGE and WB with indicated antibodies.

(C) UFL1 depletion regulates the interactions between PARP1 and MRE11 or CHK1. The stable HeLa cell lines of shNC (negative control), shUFL1 (UFL1 depleted) and FLAG-UFL1 (shUFL1-resistant UFL1 cDNA re-expressed) were lysed and subjected to PARP1 antibody and protein A agarose to immunoprecipitate PARP1 and the immunoprecipitates were analyzed by SDS-PAGE and WB with

indicated antibodies.

(D) PARP1 UFMylation regulates its interactions with MRE11. FLAG-PARP1 WT, K548R or Vector were transfected into HEK293T cells, and the cells were lysed and subjected to FLAG affinity gel to immunoprecipitate FLAG-PARP1 and the immunoprecipitates were analyzed by SDS-PAGE and WB with indicated antibodies. AZD2281: PARP1 inhibitor which was added (10 μM, 1 h) before cell harvest. IB: Immunoblot; WB: Western blotting; IP: Immunoprecipitation; HU: Hydroxyurea; Dox: Doxycycline; β-actin was used as loading controls.

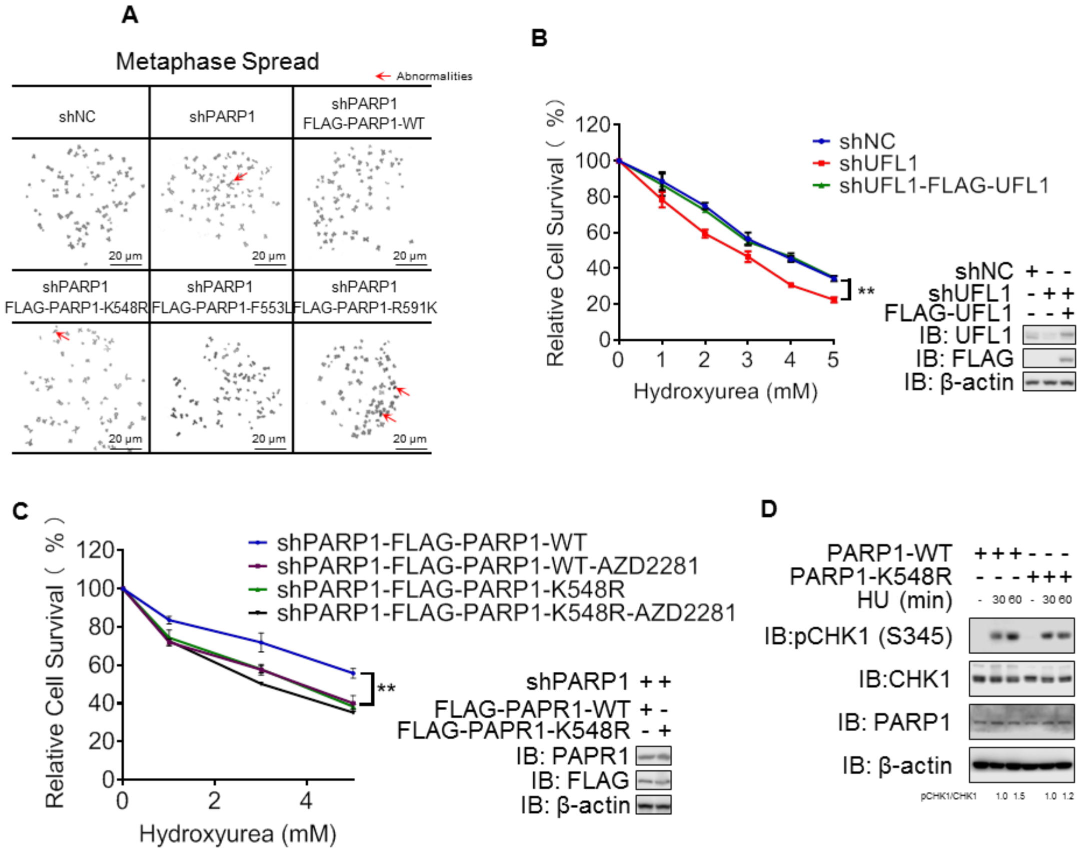

Fig. S5. PARP1 UFMylation promotes genome stability and mice viability

(A) PARP1 UFMylation deficiency decreased genome stability. The stable HeLa cell lines of shNC (negative control), shPAPR1 (PARP1 depleted) and FLAG-PARP1 (shPARP1-resistant PARP1 cDNA re-expressed) WT and mutations (K548R, F553L, R591K) were treated with nocodazole (200 ng/ml, 16 h), and then subjected to Mitotic spread analysis. Red arrow indicates abnormal chromosome. Scale bar: 20 μm.

(B) UFL1 depletion sensitized cells to replication stress. The stable HeLa cell lines of shNC (negative control), shPAPR1 (PARP1 depleted) and FLAG-PARP1 (shPARP1-resistant PARP1 cDNA re-expressed) WT and mutations (K548R, F553L, R591K, E988K) were treated with increasing concentrations of HU as indicated. The surviving colonies were analyzed after 14 days. The % surviving cells (left) and the corresponding protein expression levels determined by WB (right) are shown. Two-way ANOVA

(Tukey's multiple comparisons test): $**p<0.01$.

(C) PARP1 UFMylation deficiency sensitized cells to replication stress. HeLa cells stably expressing shNC, shPARP1 or shPARP1 with rescued PARP1-WT/K548R/F553L/R591K were treated with or without AZD2281 (1μM) before treated with increasing concentrations of HU as indicated. The surviving colonies were analyzed after 14 days. The % surviving cells (left) and the corresponding protein expression levels determined by WB (right) are shown. Two-way ANOVA (Tukey's multiple comparisons test): $**p<0.01$.

(D) PAPR1 UFMylation deficiency compromised the activation of CHK1. The MEF cells treated with or without HU (2 mM) for the indicated times were harvested. The activation of CHK1 was analyzed by SDS-PAGE and WB with indicated antibodies.

IB: Immunoblot; WB: Western blotting; HU: Hydroxyurea; Dox: Doxycycline; β-actin was used as loading controls.

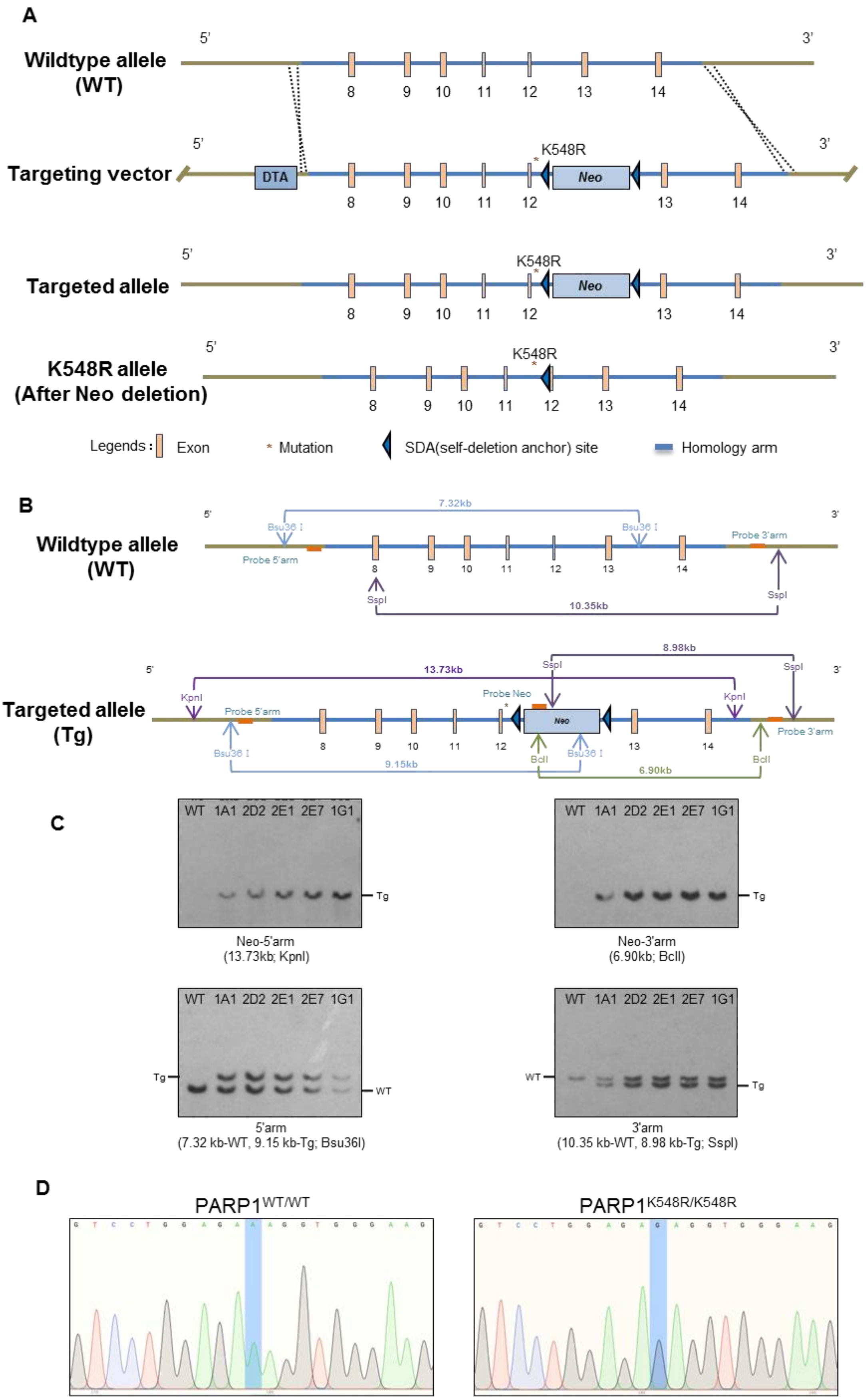

Fig. S6. PARP1 knock-in mice generation

(A) PARP1 gene targeting strategy. It includs the positions of the K548R point mutation, the neomycin resistance cassette (neo), the Thymidylate kinase (Tk) and the expected fragments in kilobases (kB) for Southern Blotting before and after successful gene targeting. Purple boxes indicate exons. Blue triangle indicates self-deletion anchor (SDA) site. Blue boxes indicate homology arm.

(B, C) Confirmation of $PARP1^{K548R}$ gene targeting. (B) Strategies for Southern Blotting. The restriction enzymes Bsu36I, SspI, KpnI, and BclI, the probes (Probe 5'arm or Probe 3'arm) and the expected fragments in kilobases (kB) for Southern Blotting before and after successful gene targeting. **(C)** Confirmation of PARP1-K548R gene targeting and CRE-recombinase-mediated neo-removal from the targeted mouse embryonic stem cells by Southern Blotting using the strategies shown in **(B)**.

(D) Sequencing of cDNA isolated from the livers of $PARP1^{WT/WT}$ and $PARP1^{K548R/\ K548R}$ littermate mice.
